# Capacity Degradation Mechanisms, Recovery Strategies, and Challenges of Flow Batteries

**DOI:** 10.1007/s40820-026-02345-y

**Published:** 2026-09-07

**Authors:** Xu Lin, Meisheng Han, Haolin Ju, Hengyuan Hu, Wanqi Hu, Jiajie Luo, Lei Wei, Lin Zeng, Wenjia Li, Tianshou Zhao

**Affiliations:** 1 Shenzhen Key Laboratory of Advanced Energy Storage, Department of Mechanical and Energy Engineering, Southern University of Science and Technology, Shenzhen, 518055 People’s Republic of China; 2 SUSTech Energy Institute for Carbon Neutrality, Southern University of Science and Technology, Shenzhen, 518055 People’s Republic of China

**Keywords:** Long-duration energy storage, Redox flow batteries, Capacity fade, Capacity recovery

## Abstract

Redox flow batteries, by virtue of their advantages in power/capacity decoupling, high safety, and long cycle life, have become a strategic support for long-duration energy storage systems; however, capacity degradation remains a bottleneck for their commercialization. This review systematically identifies the multi-dimensional mechanisms of capacity degradation: including volume imbalance caused by transmembrane migration, inactivation and decomposition of active species, electrode degradation, and valence imbalance induced by side reactions. Targeting these challenges, the review deeply explores recovery strategies from physical repair to intelligent scheduling: covering the research and development of high-selectivity membranes, precise regulation of valence states, functionalized repair of electrodes, and model-driven predictive pathways integrating digital twins. Finally, current challenges such as characterization limitations and incomplete recovery rates are summarized, and future development directions for capacity recovery are envisioned. This review aims to provide a theoretical basis and technical guidance for extending the system cycle life of flow batteries and improving capacity recovery efficiency.

## Highlights

- Systematically elucidates the physical (transmembrane migration-induced volume imbalance), chemical (inactivation of active species), and interfacial (electrode degradation, hydrogen/oxygen evolution) origins of capacity fade in redox flow batteries.
- Summarizes recovery approaches from physical repair to intelligent scheduling, including high-selectivity membranes, precise valence regulation, electrode active-site regeneration, and model-driven collaborative scheduling.
- Highlights bottlenecks (transient multi-physics coupling, kinetic limits at high current densities, stochastic predictive models) and future directions (operando multi-modal characterization, AI-accelerated smart materials discovery, digital-twin-based autonomous regulation).

## Graphical abstract

**Figure:**
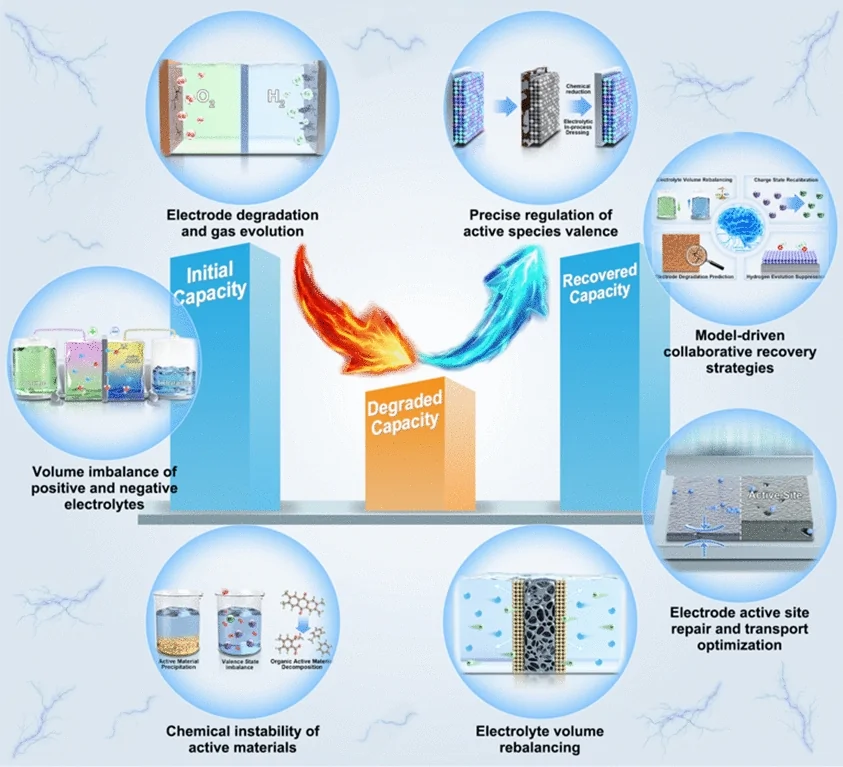

## Introduction

In the construction of new power systems, long-duration energy storage is a core facility supporting renewable energy consumption and grid security [1–3]. It can extend regulation capabilities from instantaneous balance to cross-month or even cross-season energy transfer, effectively mitigating the intermittency challenges of new energy. Under extreme conditions, long-duration energy storage can provide capacity support and inertial response, significantly enhancing grid resilience [4–7]. Furthermore, it helps reduce system costs during deep decarbonization processes and provides key support for the balance of energy supply and demand. Therefore, long-duration energy storage is an important technological foundation for achieving carbon neutrality and reconstructing the energy system [8–10]. Mechanical energy storage (e.g., pumped hydro, compressed air, gravity) is geographically constrained, has low energy density, requires large land use, and may pose ecological risks [11–13]. Fuel-based energy storage technologies (e.g., hydrogen, ammonia, and methanol) offer high energy density and the potential for cross-seasonal storage. However, their deployment is hindered by challenges including low round-trip efficiency due to multi-stage energy conversion, high storage and transportation costs, and safety concerns associated with chemical media [14–17]. By comparison, electrochemical energy storage technologies are emerging as a critical enabler of next-generation power systems, owing to their broad geographical adaptability, high energy conversion efficiency, and fast response times. Among these, lithium-ion batteries (LIBs) have become one of the dominant electrochemical storage solutions, benefiting from their high energy density and low self-discharge rate [18]. However, LIBs possess a fundamental technical shortcoming: they are prone to violent chain reactions in high-pressure or overheated environments, leading to combustion or even explosions; this safety hazard is difficult to eliminate completely in large-scale applications. In addition, LIBs are constrained by the scarcity of key mineral resources like lithium and cobalt and supply chain risks; most importantly, their storage duration is typically only 2–4 h, making it difficult to meet the demand for large-scale long-duration energy storage alone [19–21]. Therefore, to break through the limitations of existing energy storage in resource supply, safety performance, and storage duration, it is particularly urgent to develop a new generation of energy storage technology with both high safety and high efficiency.

**Fig. 1:**
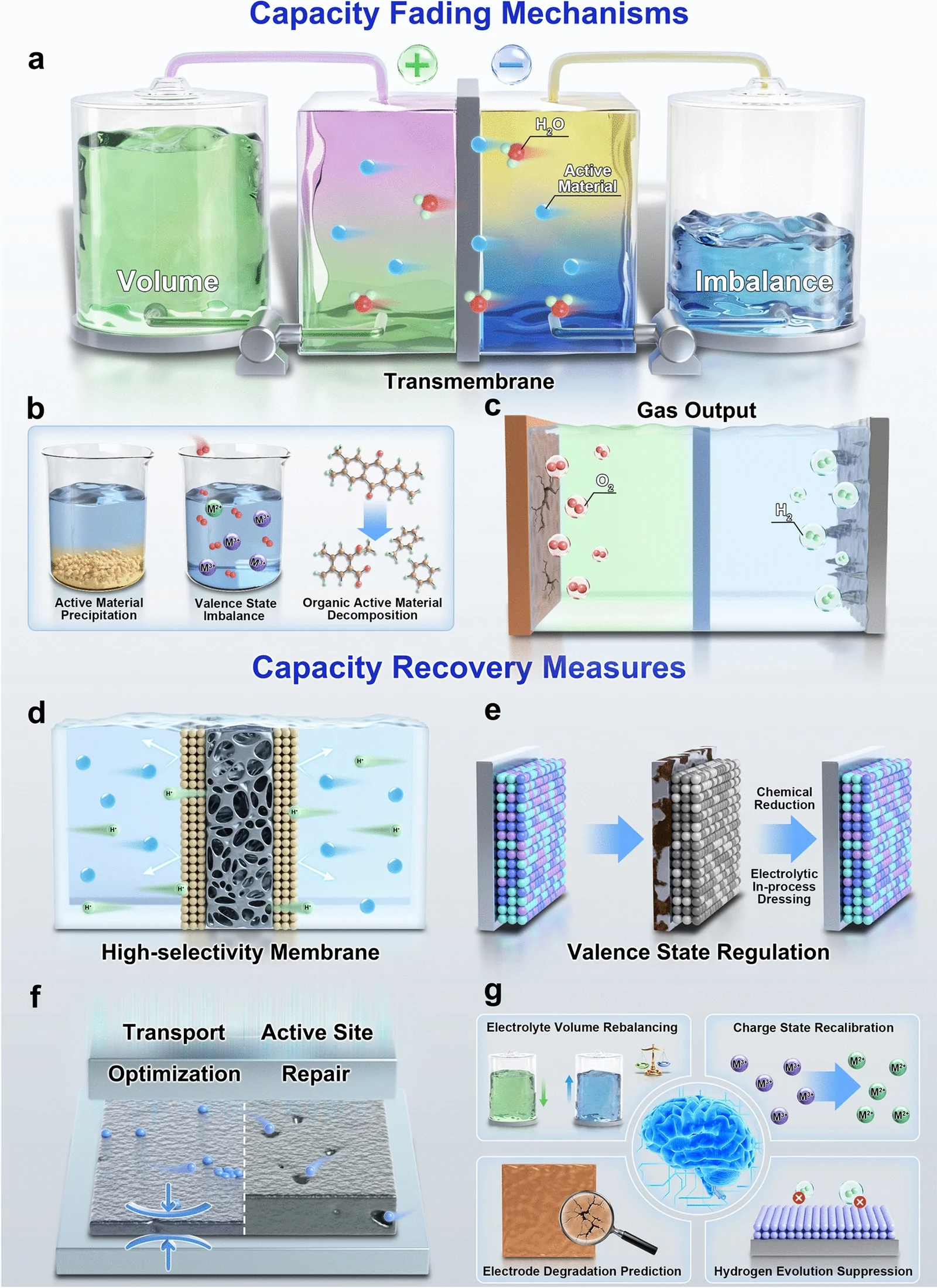
Capacity fade mechanisms of RFBs: **a** volume imbalance of electrolytes; **b** chemical instability of active materials; **c** electrode degradation and gas evolution. Capacity recovery strategies for RFBs: **d** electrolyte volume rebalancing; **e** precise regulation of active species valence; **f** electrode active-site repair and transport optimization; **g** model-driven collaborative recovery strategies

Redox flow batteries (RFBs), as a highly promising large-scale long-duration energy storage technology, play an irreplaceable key role in building a global modern low-carbon energy system [22, 23]. Their core mechanism involves using redox couples in external storage tanks, which undergo reversible electrochemical reactions at the electrodes driven by pumps [24–27]. Different from traditional solid-electrode batteries, a significant advantage of RFBs is the independent decoupling of power and capacity: Power is determined by the stack size, and capacity is determined by the electrolyte volume, providing extremely high design flexibility. Furthermore, RFB electrolytes are aqueous, possessing intrinsic safety and effectively overcoming the thermal runaway risks of traditional LIBs [28–31]. Against the backdrop of global energy structure transformation, RFBs show great application potential in grid peak shaving and long-term backup due to their high stability and low levelized cost of storage [32, 33]. While the marginal cost of increasing energy storage capacity in RFBs by simply enlarging electrolyte tanks is substantially lower than that of scaling LIB systems, RFBs have not yet achieved widespread commercialization because unavoidable capacity degradation during cycling remains a core obstacle. This review argues that the inherent economic advantage of RFBs is fully realized only when the electrolyte maintains a theoretically infinite cycle life. Capacity decay transforms the electrolyte from a stable fixed asset into a consumable, thereby undermining the levelized cost of storage advantage that forms the core competitiveness of RFBs in large-scale commercial applications. Consequently, developing effective capacity restoration strategies is of critical importance to ensure the long-term economic viability and operational stability of these energy storage systems. The capacity degradation is closely associated with degradation mechanisms related to the electrolyte. For instance, the crossover of electrolyte components can lead to volume imbalance in the half-cells, while the active materials available for redox reactions gradually diminish over repeated charge–discharge cycles. As the electrolyte accounts for ~ 50% of the total cost of a battery system, the development of effective capacity recovery strategies is of great significance for enhancing the long-term economic viability of RFBs [34]. In view of the current lack of relevant reviews, this work systematically analyzes the physical and chemical mechanisms of capacity degradation and deeply explores multi-dimensional recovery strategies. Capacity degradation is essentially a comprehensive manifestation of multi-dimensional failure mechanisms within the electrochemical system, involving physical balance destruction, chemical structure disintegration, and interface kinetic inactivation [35]. First, driven by concentration gradients, electric fields, and pressure differences, electrolyte components cross the membrane, leading to an imbalance in positive and negative electrode charges and electrolyte volumes (Fig. 1a) [36–39]. Second, the chemical instability of active species is the fundamental reason limiting the lifespan of RFBs systems. Organic systems are prone to structural degradation [40, 41]; inorganic systems face unexpected valence evolution [42–44] and precipitate formation (Fig. 1b) [45–47]. Moreover, degradation of the electrode interface, along with undesirable side reactions such as the hydrogen evolution reaction (HER) and oxygen evolution reaction (OER), constitutes another dominant factor driving capacity decay. Long-term operation leads to the evolution of the microscopic structure of the electrode surface, where the loss of active sites increases impedance; at the same time, HER/OER consumes electrons, breaks the symmetry of the state of charge, and triggers valence shifts (Fig. 1c) [48, 49]. This process restricts the long-term operational stability of the system from physical balance, chemical stability, to interface reaction kinetics.

Given that capacity degradation is an inherent phenomenon in RFBs operation, current research focuses on inhibiting the degradation process through developing active materials with both high stability and high solubility (such as water-soluble organic redox-active materials or high potential composite active materials) [50, 51]; designing membranes with high flexibility and high ionic conductivity, such as hydrocarbon-based anion exchange membranes or membranes featuring abundant sub-nanometer pores [52, 53]; and optimizing the concentration of active species, for instance by adopting a lower concentration ratio of active material to proton or by adjusting the concentration ratio between different active species [54, 55]. However, the above strategies cannot fundamentally reverse the capacity loss that has already occurred; in contrast, capacity recovery strategies can directly repair decayed capacity and achieve reversible cycling of RFBs performance. In response to the capacity degradation caused by physical transmembrane penetration, charge valence imbalance, and increased electrode polarization during long-term cycling of RFBs, researchers have proposed multi-dimensional capacity recovery strategies. First, electrolyte volume rebalancing can be achieved through high-selectivity membrane development to inhibit and correct asymmetric migration from the source (Fig. 1d). Second, active species valence can be precisely regulated by using chemical reduction or in situ electrolysis, aiming to reverse charge imbalances caused by side reactions (Fig. 1e). Third, repair of active sites and optimization of transport can be realized through electrode surface functionalization and structural improvement to reduce kinetic barriers and inhibit polarization (Fig. 1f). Last, the model-driven collaborative recovery strategies based on digital twins and optimization algorithms have emerged in recent years, achieving a transition from passive maintenance to system-level precise scheduling (Fig. 1g).

## Capacity Degradation Mechanisms

### Volume Imbalance of Positive and Negative Electrolytes

Asymmetric transmembrane transport of electrolyte components and its dynamic redistribution are the core mechanisms of physical capacity degradation during long-term RFBs cycling. This unfavorable transport behavior mainly stems from the non-ideal migration of electrolyte components such as active ions and solvent molecules under the influence of composite physical fields. On the one hand, active species will continuously cross the membrane into the opposite electrolyte under mechanisms such as diffusion caused by concentration gradients, causing irreversible self-discharge reactions and the continuous loss of effective charge carriers. On the other hand, the osmotic pressure difference generated along with ion migration will also drive the directional transmembrane transfer of water molecules, leading to a significant volume imbalance between the positive and negative electrolytes, further exacerbating capacity degradation and system instability [56–58].

**Fig. 2:**
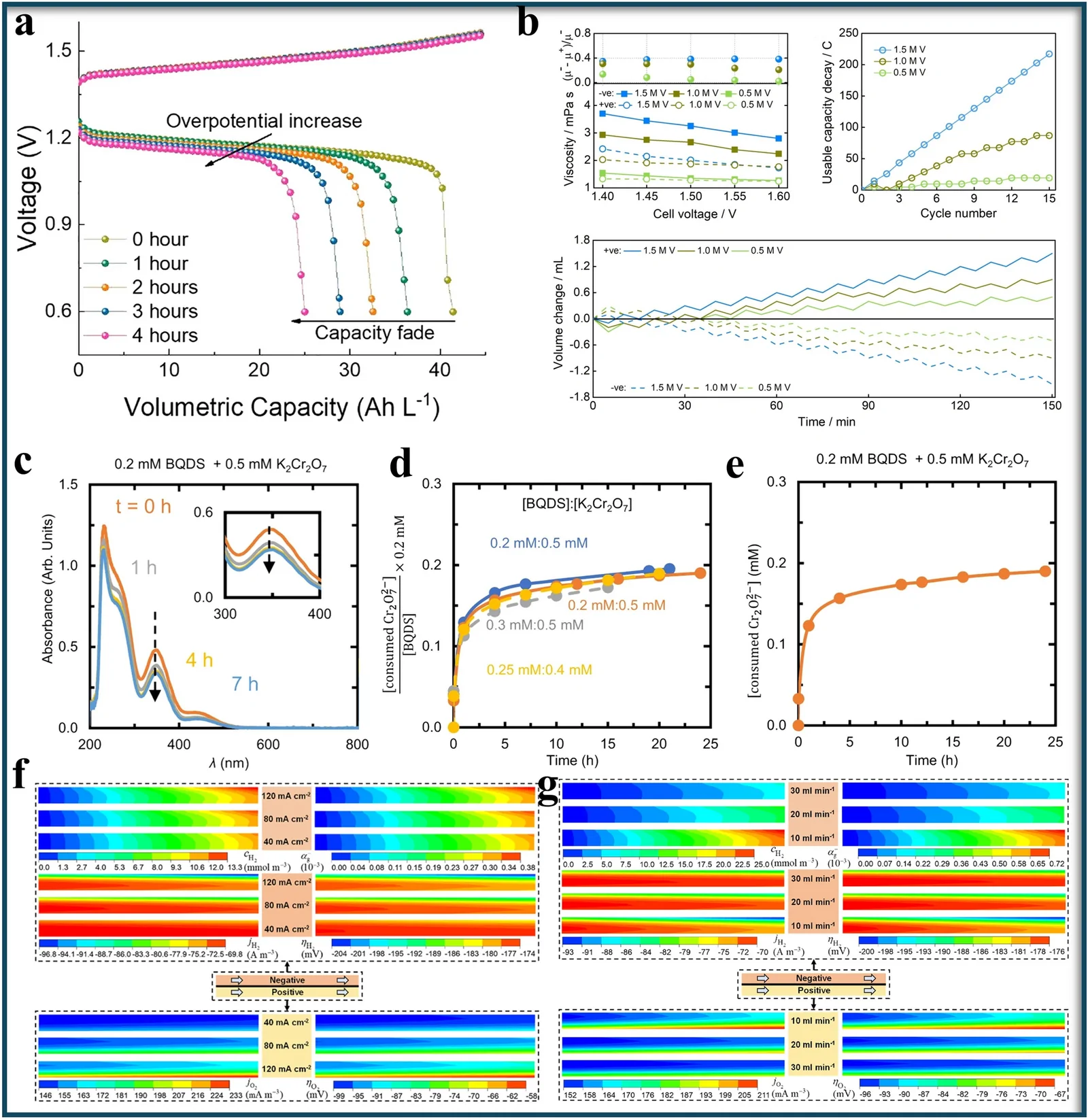
**a** Representative charge–discharge curves for 50%-SOC flow cells [59]. Copyright 2025, Elsevier. **b** Experimental analysis of mixed sulfate–chloride VFBs, including viscosity, volume changes, and capacity retention [61]. Copyright 2020, Elsevier. **c** UV–Vis spectra over time of a mixture of 0.2 mM BQDS and 0.5 mM K_2_Cr_2_O_7_ in 1 M H_2_SO_4_ [75]. **d** Calculated consumption of K_2_Cr_2_O_7_ derived from absorbance variations at 350 nm [75]. **e** Normalized K_2_Cr_2_O_7_ consumption rates across various BQDS and K_2_Cr_2_O_7_ concentrations, adjusted to a baseline BQDS level of 0.2 mM [75]. Copyright 2023, Springer Nature. **f** Spatial distributions of hydrogen concentration (c_H₂_), gas volume fraction (α̅g), activation overpotential (η_H2_, η_O2_), and volumetric current density of the gas evolution reactions (j_H2_, j_O2_) in a VFB at 50% SOC under different charging current densities (40, 80, and 120 mA cm^−2^) [84]. **g** Influence of electrolyte flow rates (10, 20, or 30 mL min^−1^) on the aforementioned gas evolution parameters at a constant charging current density of 80 mA cm^−2^ [84]. Copyright 2023, Elsevier

Mousavi et al. used Darcy’s law (Eq. 1) to calculate the hydraulic pressure (${\Delta p}_{\mathrm{porous}}$) in Zn-IRFBs, where l, μ, A, k, and Q are the length of the porous medium,

1 $$\Delta p_{{{\mathrm{porous}}}} = \frac{l}{A}\frac{\mu Q}{k}$$

the dynamic viscosity of the fluid, cross-sectional area, flow rate, and the permeability of the porous electrode, respectively. They find that when the flow rates of the positive and negative electrolytes are the same, electrolyte viscosity is the only variable affecting the hydraulic pressure of the graphite felt electrode. Subsequently, the researchers measured the electrolyte viscosity at different states of charge (SOC); the experimental results indicate that when the SOC exceeds 20%, the viscosity of the positive electrolyte shows a continuous growth trend; in contrast, the viscosity of the negative electrolyte remains basically stable after a slight initial fluctuation at 20% SOC. Therefore, the average pressure sustained by the positive electrolyte must be higher than that of the negative electrolyte. This induced pressure gradient drives the electrolyte to migrate from the high-pressure side (positive electrolyte) to the low-pressure side (negative electrolyte), carrying bound (poly)iodide active species with it. Due to the physical accumulation of (poly)iodide ions on the negative side, the positive active species lacked replenishment; the researchers investigated the capacity degradation characteristics of the battery at 50% SOC and conducted titration analysis on the negative electrolyte composition after cycling. The discharge curve shift trend (Fig. 2a) shows that, driven by severe self-discharge effects, the average capacity loss per hour of the battery is as high as 11%, and the discharge polarization is significantly enhanced. These experimental results strongly confirm the transmembrane penetration phenomenon of (poly)iodide ions: These ions migrate to the negative electrode and undergo redox reactions with the deposited zinc metal, thus becoming the main cause of capacity degradation [59]. For all-vanadium flow batteries (VFBs), Song et al. analyzed the viscosity characteristics of the positive and negative electrolytes under different SOC through experimental analysis. The results show that as the vanadium concentration increases from 1.1 to 1.7 M, the overall viscosity of the electrolyte increases significantly. Simultaneously, as the SOC increases, the viscosity of both positive and negative half-cells shows a downward trend, and at the same temperature, the negative electrolyte always maintains a higher viscosity level than the positive one. This viscosity difference drives the electrolyte to continuously migrate from the negative to the positive side under the influence of osmotic pressure gradients and gradually accumulate, leading to volume imbalance on both sides. Because the negative electrolyte capacity is relatively insufficient, the actual available capacity of the VFB is limited, thereby causing capacity degradation. The researchers subsequently verified the influence of flow rate; experimental comparison finds that when the flow rate decreases from 40 to 20 mL min^−1^, the slope of the volume change curve tends to level off, indicating that the transmembrane migration rate slows down. This is mainly because the decrease in flow rate reduced the pressure difference between the positive and negative half-cells, thereby inhibiting the transmembrane migration of the electrolyte, helping to alleviate volume imbalance and slow down the rate of capacity degradation [60]. To further understand the capacity degradation mechanism of more types of RFBs, Song et al. further analyzed the volume transfer phenomenon of six different RFBs. To exclude the influence of supporting electrolytes, the researchers first conducted experiments using a sulfate–chloride binary mixed acid as the supporting electrolyte. Through the measurement of the electrolyte viscosity of the positive and negative half-cells, Fig. 2b shows that under different battery voltages and three vanadium concentration conditions, the viscosity of the negative electrolyte is always higher than that of the positive one. Although this study adopted a mixed acid supporting electrolyte, the viscosity change laws of the two types of VFBs are still similar. In addition, the experiment measured that at vanadium concentrations of 0.5, 1, and 1.5 M, the positive electrolyte volume increases by 0.5, 0.9, and 1.3 mL, respectively, a change trend is consistent with conventional VFBs. After 15 cycles, the capacity degradation values of the above three systems are 19.3, 86.84, and 217.1 C, respectively. Additionally, Fe-V RFBs also show the same volume transfer trend. However, in V-Fe RFBs and all-Fe RFBs, because the viscosity of the positive electrolyte is greater, the volume migrates to the negative side. Cycle test results indicate that in 15 charge–discharge cycles, the net transfer of Fe–Cr RFBs electrolyte with similar viscosity was zero, which further confirmed the electrolyte migration mechanism proposed earlier [61].

Besides, a decisive tactical distinction must be established between transient fluidic imbalances and permanent barrier degradation to determine the appropriate engineering intervention at the end of this analytical section. On the one hand, macroscopic capacity fluctuations driven primarily by osmotic pressure disparities or localized concentration gradients represent fluidic volume asymmetries that can be completely rectified online through routine physical electrolyte remixing or automated hydraulic rebalancing line reversals. On the other hand, structural cross-contamination caused by membrane pore expansion or severe chemical degradation of the polymeric matrix represents a fundamental barrier failure that remains entirely unresponsive to simple fluidic volume adjustments. Mitigating these permanent active species migration fluxes demands comprehensive membrane redesign strategies that focus on tightening sub-nanometer ion transport channels or introducing localized electrostatic repulsive charges to suppress crossover at its material origin.

### Chemical Instability of Active Materials

During the long-term cycling process of RFBs, the physical and chemical stability of active materials is the core factor determining the capacity retention rate, among which precipitation, oxidation, and decomposition of electrolyte active species are recognized as the most important reasons for inducing capacity degradation. Specifically, thermal precipitation of active species (such as VO_2_^+^) at high temperatures will directly reduce the total amount of active species participating in reactions [62]; some low-valence active species are susceptible to air oxidation, leading to electrolyte SOC imbalance [63]; while the structural decomposition of active molecules in organic or polyiodide systems will cause permanent inactivation of the species [64]. These irreversible chemical changes lead to a decrease in the concentration of active species participating in redox reactions, thereby causing capacity degradation. Below, the review will provide a detailed introduction from the following perspectives: precipitation, adverse redox reactions and decomposition.

#### Precipitation

Precipitate formation directly leads to RFBs capacity degradation by consuming the concentration of active species and physically blocking flow channels and reaction interfaces [65]. For VFBs, at elevated temperatures the hydrated pentavalent vanadium ions in their native state exist as stable octahedral VO_2_^+^ complexes coordinated with three neutral water molecules. Thermal activation drives the progressive deprotonation of these chemically bound water molecules forcing the sequential transformation of the localized coordination sphere into highly reactive neutral VO(OH)_3_ intermediates. These deprotonated neutral species rapidly undergo continuous olation and oxolation condensation reactions where bridging oxygen networks continuously extend to form dense polymerized chemical strands. This uncontrolled structural cross linking fundamentally culminates in the irreversible nucleation and subsequent precipitation of crystalline vanadium pentoxide solids directly reducing the active chemical inventory of the positive reservoir. Vijayakumar et al. used nuclear magnetic resonance (NMR) to characterize VFBs electrolytes and find that at room temperature, VO_2_^+^ exists as a stable hydrated coordinated ion [VO_2_(H_2_O)_3_]^+^, but when the operating temperature rises, this ion transforms into a neutral H_3_VO_4_ molecule through a deprotonation process and subsequently generates irreversible V_2_O_5_ precipitates through a hydroxyl condensation reaction. The formation of precipitates directly reduces the concentration of active species, leading to a substantial capacity degradation [66]. Furthermore, Laurent Cassayre et al. characterized the resulting solid phases through thermogravimetric analysis, X-ray diffraction (XRD), and scanning electron microscopy (SEM). Analysis shows that the dissolved V^2^⁺, V^3^⁺, and VO^2^⁺ ions in the solution all precipitate as hydrated crystals, with their precipitate products being hydrates of VSO_4_, V_2_(SO_4_)_3_, and VOSO_4_, respectively. These precipitate products, like V_2_O_5_, all have an adverse impact on VFBs capacity [67]. Li et al. found that after the first discharge of V-Mn RFBs, a brown precipitate was formed on the inner wall of the electrolyte tank; by the 10th discharge, the amount of precipitate increased significantly. However, the amount of precipitate began to decrease after the 25th cycle and basically disappeared at 50th cycle. Through XRD analysis, the composition of the precipitate is confirmed as MnO_2_. The above results indicate that the MnO_2_ precipitate existing in the positive electrolyte tank after discharge originates from the disproportionation reaction of Mn^3+^ ions generated during the charging process with water in the electrolyte to convert into MnO_2_, which failed to be completely reduced to Mn^2+^ ions during the discharge stage. This part of the MnO_2_ precipitate cannot participate in subsequent redox reactions, thus leading to a decrease in battery capacity [68]. In addition to these two types, other RFBs will also inevitably generate precipitates under certain specific conditions, affecting the concentration of active species participating in redox reactions, thereby reducing RFBs capacity.

#### Unfavorable Redox and Decomposition

In RFBs electrolytes, the occurrence of irreversible or unintended redox reactions of active species is an important cause of RFBs capacity degradation. In conventional RFBs systems, it is difficult to completely isolate active species from the air, making them easily oxidized by oxygen in the air, resulting in a decrease in the concentration of active species available for normal redox reactions [69]. Clarifying the specific mechanism of this oxidation process is a key prerequisite for effectively inhibiting RFBs capacity degradation. Shin et al. investigated the oxidation mechanism of iron and 2, 2-bis(hydroxymethyl)-2,2′,2″-nitrilotriethanol (Fe(BIS–TRIS)) complex active species using ultraviolet–visible (UV–Vis) absorption spectroscopy. The experiment observes a decrease in the absorbance peak intensity at 916 nm and an increase in the peak intensity at 467 nm. It is known that the 916-nm peak belongs to charged-state Fe(BIS–TRIS), while the 467-nm peak corresponds to its discharged state. The above changes indicate that charged-state Fe(BIS–TRIS) undergoes spontaneous oxidation upon contact with air, a self-discharge process occurs, specifically manifested as an enhancement of the discharged-state characteristic peak and a weakening of the charged-state characteristic peak. This result confirms that the oxidation (discharge) of charged-state Fe(BIS–TRIS) originates from its spontaneous reaction with oxygen in the air. Subsequently, the researchers conducted experiments under nitrogen conditions isolated from oxygen, and comparison finds that iron-based RFBs running in an air environment exhibit rapid capacity degradation, with the capacity retention rate dropping to 5.3% after only 20 cycles. Under nitrogen protection, the oxidative degradation of active species is significantly inhibited, and the battery capacity retention rate can reach 96.0% after 100 cycles, with the Coulombic efficiency (CE) stable at approximately 99.6%. This result further indicates that the oxidation reaction triggered by oxygen is the main factor in the capacity degradation of this system [70]. In addition, under specific conditions, if active species undergo an unintended reduction reaction, it will also cause a decrease in effective active species concentration, thereby affecting the capacity stability of the RFBs. Yang et al. systematically studied the capacity degradation mechanism of iron-based RFBs using iron-cyanide complexes (Fe(CN)_6_) as positive active species and iron-sulfonated triethanolamine complexes (Fe(DIPSO)) as negative active species. The researchers proposed that capacity degradation originates from side reactions between Fe(CN)_6_^3−^ and free ligands and the corresponding mechanism is analyzed. Specifically, the excess DIPSO ligand in the negative electrolyte is mainly used to stabilize the Fe(DIPSO) complex. During battery cycling, this free ligand can penetrate the cation exchange membrane into the positive electrode in an alkaline environment and reduce Fe(CN)_6_^3−^ to Fe(CN)_6_^4−^. UV–Vis spectroscopy analysis shows that Fe(CN)_6_^4−^ continuously accumulates in the positive electrolyte, and the color change of the negative electrolyte also correspondingly shows the generation and accumulation of Fe^2+^(DIPSO). This unbalanced product accumulation leads to capacity imbalance, thereby causing irreversible capacity degradation [71].

The intrinsic chemical instability of organic redox-active molecules and their irreversible decomposition during cycling (such as hydrolysis, dimerization, or radical degradation) cause unfavorable capacity degradation of aqueous organic redox flow batteries (AORFBs) by continuously consuming the active concentration of effective charge carriers. First, under highly alkaline conditions, universal nucleophilic attack driven by aggressive hydroxide ions targets electrochemically active carbonyl centers or vulnerable quinone aromatic rings, inducing irreversible ring opening or irreversible hydroxylation. Mitigating this pathway requires molecular engineering strategies that introduce strong electron-donating groups, such as hydroxyl or amino groups, to elevate local electron density and repel nucleophilic reactants via electronic shielding. Second, transient radical anions generated during single-electron transfer interludes frequently undergo irreversible radical-coupling dimerization due to excessive localized spin density. Suppressing this dimerization vector demands expanding the aromatic core conjugation framework or grafting terminal sulfonate groups to facilitate pristine charge delocalization across the molecular skeleton. Third, reactive organic cores bearing alpha–beta-unsaturated carbonyl groups are exceptionally susceptible to devastating Michael addition reactions. Defending against these addition cascades requires deploying strategic steric-hindrance obstacles, such as bulkier alkyl chains or localized methyl blockers, to permanently seal the active addition coordinates and preserve long-term structural integrity [72–74]. Modak et al. analyzed the decomposition of oxidized 4,5-dihydroxy-1,3-benzenedisulfonic acid (BQDS) using spectroscopy and statistical reasoning techniques. Previous studies indicate that oxidized BQDS is prone to self-discharge reactions with water, decomposing to form a series of hydroxyl-substituted para-hydroquinone species with redox potentials lower than BQDS. To verify this conclusion, the researchers used K_2_Cr_2_O_7_ as an oxidant and performed the UV–Vis absorption spectrum of a 0.5-mM K_2_Cr_2_O_7_ solution after adding 0.2 mM BQDS (Fig. 2c) and find that the spectral intensity decreases uniformly, which is consistent with the decrease in K_2_Cr_2_O_7_ concentration. Subsequently, according to the standard curve (Fig. 2d), the decline of the absorption peak at 350 nm is tracked and used as a monitoring indicator for K_2_Cr_2_O_7_ concentration (Fig. 2e), and it is further found that after adding BQDS, the K_2_Cr_2_O_7_ concentration immediately decreases by 0.07 mM, which strongly confirms the oxidative decomposition of BQDS [75]. Volodin et al. analyzed the decomposition of ferrocene-based FPMAm-co-METAC polymer in zinc-based RFBs. Electrospray ionization mass spectrometry (ESI–MS) analysis reveals the detachment of its hydrolysis product, (2-hydroxyethyl)trimethylammonium chloride solubilizing units, from the polymer backbone. The reversible condensation-hydrolysis reaction of ester bonds in the polymer solubilizing units, and the subsequent migration of (2-hydroxyethyl)trimethylammonium chloride units to the negative electrolyte, are the main reasons for capacity degradation [76]. Xu et al. found that AORFBs using ferrocene/naphthalimide-based ionic bipolar redox-active organic molecules as active species exhibits rapid capacity degradation after 140 cycles. The researchers subsequently confirmed using high-resolution mass spectrometry (HRMS) that the accelerated capacity degradation originates from the breaking of specific chemical bonds (such as those between ferrocene groups and quaternary ammonium connecting groups) within the molecule. This structural disintegration not only leads to the inactivation of electroactive centers, but the generated fragmented products will further produce solid precipitates due to significantly reduced solubility, physically destroying the uniformity of the electrolyte [77]. The above studies show that the capacity degradation of AORFBs mainly stems from the chemical instability of organic active molecules themselves. Therefore, enhancing the intrinsic stability of functional groups through molecular structure design has become the core pathway for inhibiting side reactions and delaying capacity degradation.

### Electrode Degradation and Gas Evolution

Electrode degradation is an important cause of RFBs capacity degradation. Electrode materials may undergo corrosion, passivation, or loss of active sites during long-term cycling, reducing reaction kinetic performance [78, 79]. Singh et al. found that at a current density of 80 mA cm^−2^, after 100 cycles, the total charge–discharge duration of a VFB significantly decreases from 944 to 522 s. This capacity degradation is closely related to the intensification of battery polarization: The voltage difference between the end of charging and the start of discharging in the first cycle is 0.636 V, while it rises to 0.733 V after 100 cycles. An increase in voltage polarization means a reduction in electrode active sites, degradation of electrolyte performance, or an increase in membrane ohmic impedance, making the battery to reach the cut-off voltage earlier during constant current charge–discharge, finally manifested as shortened effective charge–discharge time and capacity loss. The researchers subsequently performed Raman spectroscopy analysis on the degraded positive electrode after 100 cycles, which shows that the defect signal intensity of the carbon fibers is significantly higher than the surrounding graphitized areas, which may be related to the higher oxidation sensitivity of amorphous carbon fibers. Furthermore, the violent oxidation process during cycling changes the composition of oxygen-functional groups on the carbon paper surface: from initially being dominated by hydroxyl groups (–OH) to a mixed state where carboxyl groups (–O–C=O) and hydroxyl groups coexist, and this surface chemical change finally accelerates the degradation of the electrode [80].

In various battery systems, HER and OER are ubiquitous and are the important reasons causing efficiency decline and capacity degradation. Among them, HER triggers charge (valence) imbalance between the positive and negative electrolytes by competitively consuming electrons during the charging process, causing the degree of reduction of the negative active species to lag behind that of the positive one, fundamentally causing continuous degradation of the available capacity of the RFBs. Fundamentally, parasitic HER and OER are rooted in the thermodynamic mismatch between active redox potentials and the electrolyte electrochemical stability window (0.00 and 1.23 V vs. standard hydrogen electrode (SHE), respectively). In VFBs, the negative V^2+^/V^3+^ potential (ca. − 0.26 V vs. SHE) closely approaches the HER onset potential, making HER readily induced under low pH and high overpotential conditions and aggravating state-of-charge asymmetry. For Zinc-based systems, the Zn deposition/dissolution potential (ca. − 0.76 V vs. SHE) is significantly lower than the HER threshold; thus, despite the high kinetic overpotential (passivation) of specific substrates, HER remains the dominant parasitic side reaction. In contrast, iron-based systems face self-catalytic degradation because the electrodeposited Fe (− 0.44 V) intrinsically acts as an HER electrocatalyst, triggering destructive pH drift. On the positive side, while equilibrium potentials of mainstream couples (e.g., VO^2+^/VO_2_^+^ at + 1.00 V) remain safe, severe localized concentration polarization during high-rate charging, or operations under specific ligand or pH conditions, can force transient potential excursions beyond 1.23 V that render OER a critical degradation pathway, causing subsequent oxidative mass loss of carbon current collectors [81–83]. Qian et al. constructed a transient multi-dimensional multi-physics VFBs model based on coupling multiphase material transport and electrochemical reactions, which indicates that HER occurs continuously throughout the charge–discharge process due to its negative activation overpotential. Figure 2f shows that as the current density increases from 40 to 120 mA cm^−2^, the hydrogen concentration at the negative outlet increases from 10.31 to 11.56 mmol m^−3^. Studies indicate that HER is mainly concentrated in the area close to the bipolar plate, which mainly originates from the large electrochemical potential difference between the graphite electrode and the liquid electrolyte at this location, thus forming a high local potential. Figure 2g shows that HER gradually enhances along the flow direction, the reason is that during the charging process, the SOC of the electrolyte continuously increases along the flow direction, leading to charge imbalance. OER only appears at the end of charging. The study further finds that when the operating temperature rises from 298.15 to 313.15 K, the HER reaction rate increases by nearly seven times from 163.3 to 1167.1 A m^−2^. The influence of temperature on OER activation overpotential is more significant than that on HER. Furthermore, HER exacerbated by rising SOC leads to a 0.54% SOC imbalance after 10 cycles, accounting for 11% of the total capacity loss [84].

In summary, the above analysis covers the main capacity decay mechanisms of most current RFBs. However, the dominant mechanisms of capacity degradation vary significantly across different RFB categories. VFBs are primarily influenced by transmembrane crossover driven by the multi-valence characteristics of vanadium ions, which leads to volume and valence state imbalances between the positive and negative electrolytes. In contrast, the lifespan of AORFBs is mainly limited by the chemical structural decomposition of organic redox-active molecules, involving processes such as hydrolysis or dimerization. Meanwhile, zinc-based RFBs are critically challenged by the accumulation of dead zinc and electrode degradation resulting from uncontrollable dendrite growth. Crucially, the fundamental failure modes of RFBs do not operate in thermodynamic isolation but rather trigger profound cross-scale ripple effects, in which chemical degradation directly dictates altered physical transport phenomena. Specifically, the progressive localized chemical precipitation of active materials or molecular decomposition solids directly clogs the interconnected microscale pore networks within the carbon fiber matrix. This local structural blockage induces a severe, nonuniform reduction in the intrinsic permeability of the porous electrodes, thereby restricting local fluidic pathways. Consequently, fluid velocity vectors reorganize chaotically, amplifying localized hydraulic pressure differences and generating intense concentration gradients across the membrane phase boundary. These amplified physical driving forces systematically accelerate the parasitic transmembrane mass crossover of active ionic species across the separator, thereby fundamentally destabilizing the balanced volumetric inventory of the negative and positive reservoirs. Subsequently, this accelerated physical crossover feeds directly back into the interfacial domain, establishing an aggressive multi-physics degradation loop under continuous cycling. The severe stoichiometric ion imbalance and localized concentration polarizations caused by crossover gradients induce massive deviations in local equilibrium potentials, triggering severe overpotential spikes within the restricted flow zones. When the local electrode potential exceeds the stable thermodynamic window of the supporting aqueous phase, it immediately accelerates destructive side reactions, including OER and HER. Concurrently, these extreme interfacial overpotentials significantly intensify the electrochemical oxidative corrosion of pristine carbonaceous fibrous substrates, leading to loss of functional active sites and surface structural collapse. This cascade of interconnected degradation vectors transforms temporary chemical variations into permanent multiphase material damage, demonstrating that single-cell longevity is strictly bound by these cumulative cascading degradation networks.

Besides, researchers have verified various capacity degradation mechanisms of RFBs through a series of characterization methods, such as using NMR, UV–Vis, ESI–MS, and HRMS to track the oxidative decomposition pathway and chemical instability of active species; using XRD, SEM, etc., to analyze the precipitation behavior and phase characteristics of active species; using Raman, etc., to reveal the oxidative degradation, functional group evolution, and loss of active sites on the electrode surface; and using titration analysis, viscosity/volume measurement, and multi-physics numerical simulation to quantify the dynamic influence of transmembrane migration, volume shift, and gas evolution side reactions. However, existing characterization methods mostly belong to offline testing or postmortem analysis, which are difficult to fully capture the transient changes during the operation process and deeply understand the complex capacity degradation mechanisms. Therefore, there is an urgent need to develop new in situ characterization technologies to capture in real time the dynamic evolution process of active species, electrode interfaces, and electrolyte systems during battery operation, thereby systematically revealing the degradation mechanism under multi-field coupling on temporal and spatial scales. This will lay a theoretical and technical foundation for the practical realization of RFBs systems with minimal capacity degradation and long cycle life.

## Capacity Recovery Strategies

### Electrolyte Volume Rebalancing

**Fig. 3:**
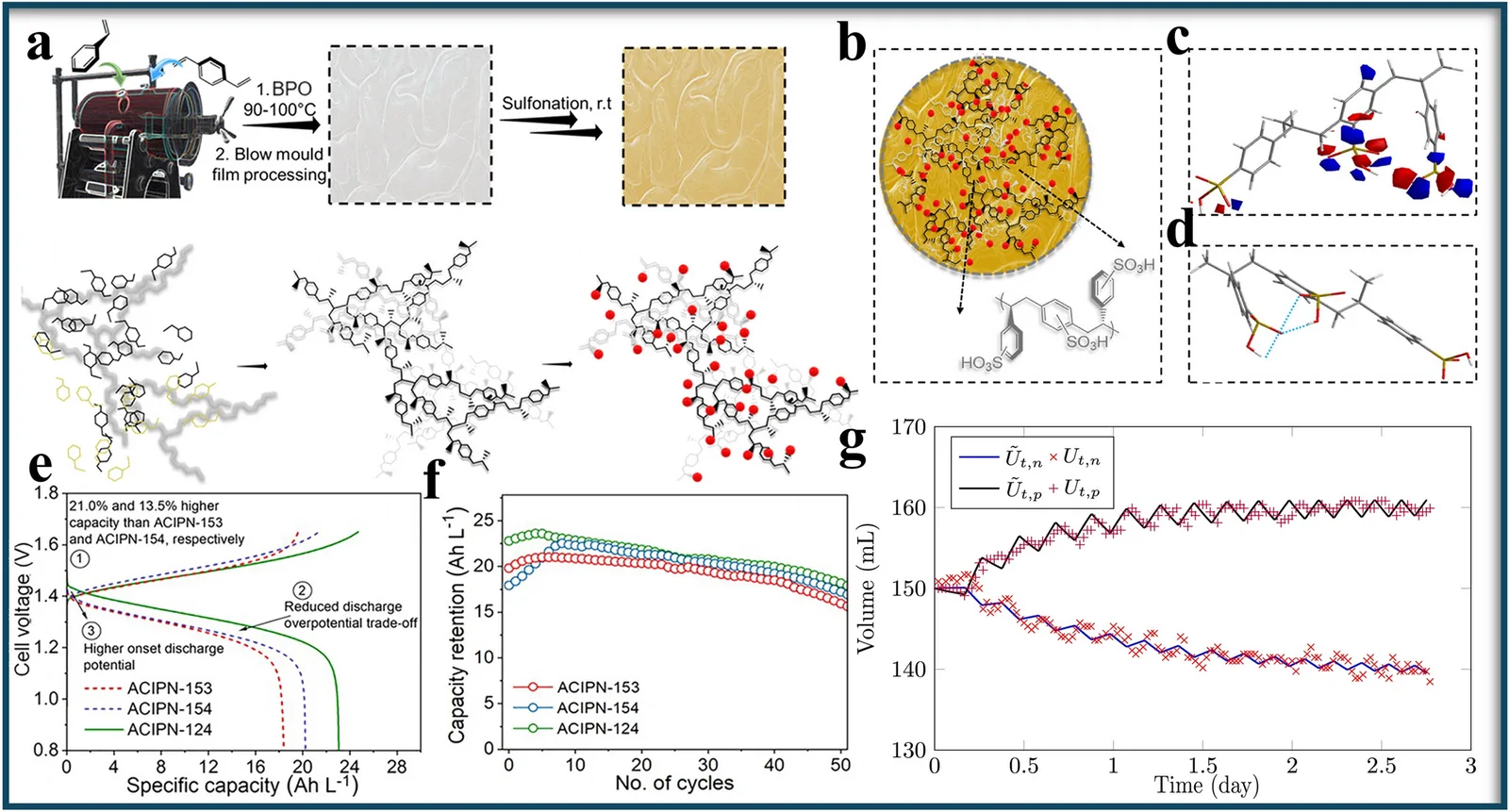
**a** Synthetic route for large-scale polyethylene-based melt-interpenetrating proton exchange membrane [87]. **b–d** Chemical structures and iso-structural configurations of the sulfonated copolymer repeating units within the interpenetrating network [87]. **e** Improvements in galvanostatic charge–discharge (GCD) results of thickness-optimized ACIPN-124 in comparison with ACIPN-153 and ACIPN-154 at 100 mA cm^−2^ [87]. **f** Comparative capacity maintenance and post-cycling analysis of ACIPN-x membranes over 100 GCD cycles [87]. Copyright 2024 American Chemical Society. **g** Measured and estimated electrolyte volume changes of both half-cells during the charging/discharging cycling experiment for Nafion-115 membrane [88]. Copyright 2021, Elsevier

Non-equilibrium transmembrane migration (driven by concentration gradients and convection) is the primary cause of electrolyte imbalance and capacity degradation [85]. In response to the above issues, hydraulic and remixing strategies serve only as external physical compensation approaches and cannot fundamentally avoid the volume imbalance and cross-contamination of the electrolyte. Therefore, only by developing high-selectivity membranes or optimizing their physical structure to precisely inhibit the asymmetric penetration of active species, system capacity can be effectively restored, achieving a high-specific-capacity recovery rate. Specifically, natively within conventional porous separators unconstrained hydrated active ions and free water molecules driven by high concentration gradients easily diffuse through large amorphous hydrophilic domains. In stark contrast, advanced high-selectivity membranes feature highly tailored sub-nanometer ionic transport channels. These customized paths possess pristine pore sizes precisely positioned between the tight hydrodynamic diameters of small proton carriers and the significantly larger effective solvation radii of multi-valence active metallic complexes or bulky organic redox centers. Consequently, protons or supporting carrier ions hop seamlessly through the confined hydrated domains while the larger reactive species are geometrically blocked at the membrane phase boundary, fundamentally eliminating mass transport-driven volume asymmetric drift [86]. Jeet et al. synthesized a low-cost, anti-permeation poly(styrene-*co*-divinylbenzene)-interpenetrated polyethylene membrane by blow mold extrusion of as-synthesized interpenetrating network-type pallets obtained from free-radical copolymerization and melt interpenetrating reactions of styrene, divinylbenzene, and polyethylene (Fig. 3a). The researchers named this membrane based on anionic cross-interpenetrating network (ACIPN) in the following way: In ACIPN-153, 150 represents the membrane thickness of 150 μm, and 3 represents a sulfonation time of 3 h. To ensure that the membrane has excellent proton adaptability, the researchers analyzed the matrix phase distribution of the membrane (Fig. 3b), and find that the sulfonation of this interpolymer membrane has been achieved. Surface structure analysis indicates that the polymer backbone does not show obvious ionic charge characteristics (Fig. 3c), while the hydrogen bonding interactions existing between the sulfonic acid groups in the chain make it show high ionizability to protons (Fig. 3d). Comparison finds that ACIPN-124 shows superior discharge performance to ACIPN-153 and ACIPN-154 at a high current density of 100 mA cm^−2^. Its specific capacity increases by approximately 12% (Fig. 3e), and the onset discharge voltage reaches 1.44 V, higher than ACIPN-153 (1.40 V) and ACIPN-154 (1.41 V). Furthermore, RFBs composed of this membrane maintain excellent specific capacity retention (> 85%) in 50 charge–discharge cycles (Fig. 3f). Performing a rebalancing operation after discharge, the average capacity degradation per cycle is only 0.6%, and the capacity can be restored from 3 to 23 Ah L^−2^, with a recovery rate of about 98%. These results indicate that the ACIPN-x series of membranes have stable electrochemical performance, can be used for VFBs systems using stable electrolytes, and support their long-term operation. Crucially, from the perspective of extended operation, this physical rebalancing approach paired with the ACIPN membrane introduces no external chemical reagents, thereby completely eliminating the risk of foreign by-product accumulation that could alter the rheological or transport properties of the bulk electrolyte. Furthermore, the post-recovery secondary degradation rate remains highly linear and predictable at approximately 0.6% per cycle, demonstrating that periodic physical restoration does not trigger accelerated capacity fade over consecutive cycles and ensures robust long-term systemic stability [87]. Li et al. developed a model-based method to determine the permeability characteristics of membranes in VFBs systems, including vanadium ion crossover and water transfer behavior, and estimated the vanadium ion permeability and water transport coefficients through nonlinear optimization combined with the measured negative and positive half-cell potentials. This model can significantly simplify the characterization of membrane permeability characteristics in traditional methods. As shown in Fig. 3g, the electrolyte volume of the positive cell gradually increases and finally stabilizes at around 160 mL, while the volume of the negative cell correspondingly decreases and stabilizes at approximately 140 mL, which is completely consistent with currently confirmed conclusions. This model can be applied to predict capacity degradation of commercial RFBs systems based on changes in membrane permeability and arrange maintenance measures to recover capacity [88].

In summary, by developing high-performance ion exchange membranes and constructing precise physical penetration models, the asymmetric migration of active species can be significantly inhibited from the source, and scientific guidance can be provided for periodic hydraulic maintenance. Experiments show that optimized membranes combined with volume rebalancing operations can achieve a physical capacity recovery rate of as high as 98%, effectively solving the long-term cycle capacity degradation problem caused by hydraulic imbalance. However, the current challenge is that a single physical barrier or post-repair strategy is difficult to completely eliminate the transmembrane penetration of active species, etc.; therefore, there is an urgent need to develop membranes that can more effectively block the migration of active species.

### Precise Regulation of Active Species Valence

Precisely regulating the valence of active species is a key pathway for reversing charge imbalance and recovering the lost capacity of RFBs. By means of chemical reduction, potential control, or introducing specific catalytic conversion mechanisms, active species can be readjusted to an ideal equilibrium state, effectively offsetting the charge loss caused by side reactions such as HER and air oxidation, thereby reviving the dysfunctional active materials in the system and significantly improving the capacity recovery rate [89, 90]. Currently, research in this field is mainly concentrated on VFBs, which have the highest degree of commercialization. For this reason, the following will carry out a systematic discussion from two parts: first, the relatively mature valence regulation strategies in VFBs; second, the research progress of related repair methods in emerging RFBs such as zinc-based and organic systems.

#### VFBs

**Fig. 4:**
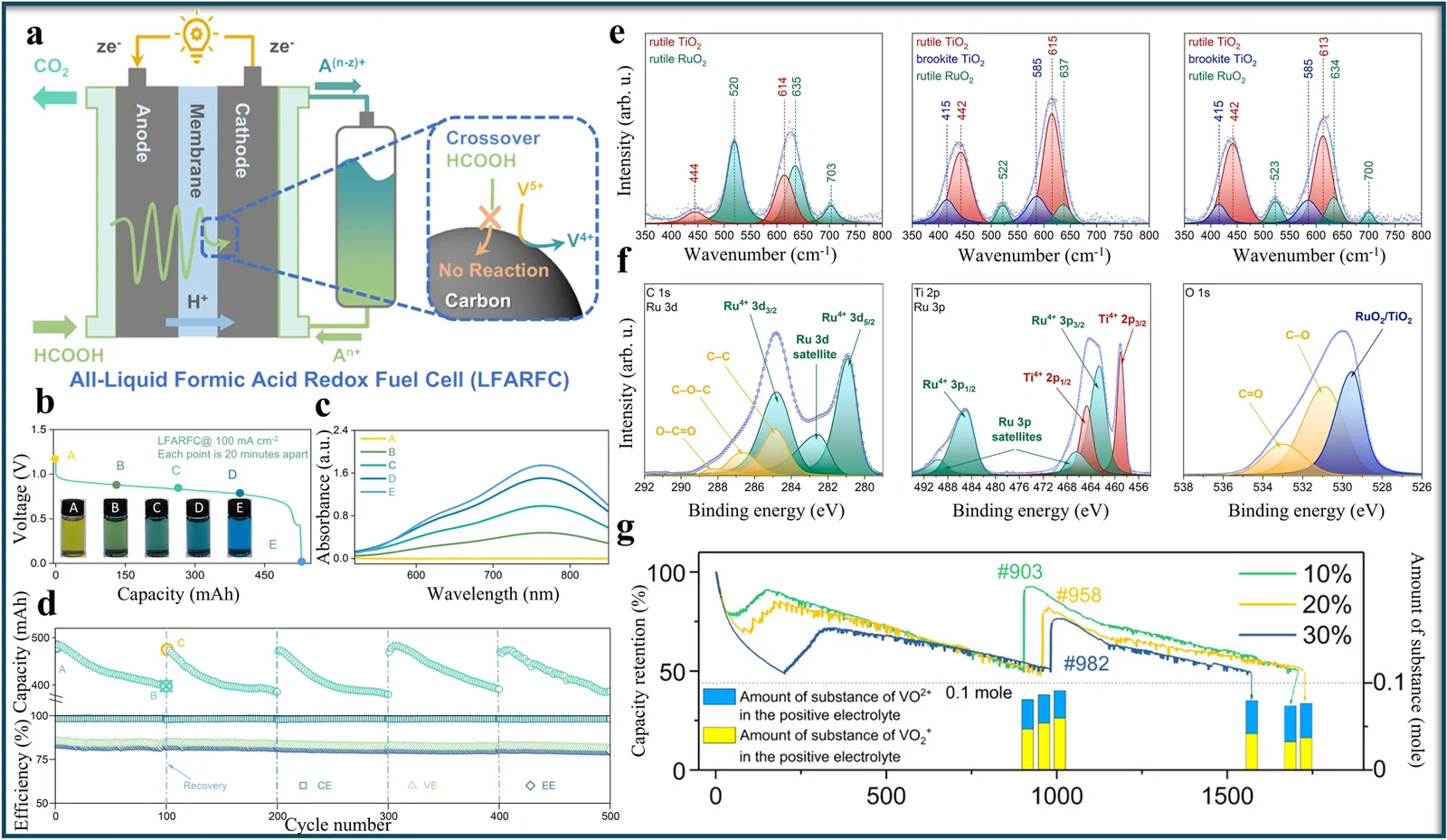
**a** Schematic representation and underlying reaction mechanism of the LFARFC [93]. **b** Process of electrolyte rejuvenation within LFARFCs [93]. **c** UV–Vis curves of the variation of vanadium electrolytes [93]. **d** Capacity recovery test [93]. Copyright 2024, Royal Society of Chemistry. **e** Deconvolution of Raman spectra from various areas of the RuO_2_/Ti electrode [96]. **f** High-resolution XPS spectra of the RuO_2_/Ti electrode surface [96]. Copyright 2023, Elsevier. **g** Comparative capacity maintenance and positive electrolyte speciation for VFBs systems with varying concentrations (− 10%, − 20%, and − 30%) after the first cycle [97]. Copyright 2020 American Chemical Society

In VFBs, side reactions like air oxidation and HER cause the average electrolyte valence to shift toward higher states, leading to continuous capacity degradation [91, 92]. Researchers have conducted many studies to solve this problem. Wei et al. restored RFBs capacity with the help of an all-liquid formic acid redox fuel cell (LFARFC); the LFARFC principle is shown in Fig. 4a, mainly using liquid redox species as the cathode fuel; the redox reaction of its cathode cell is as follows: high-valence redox ions $\left({\mathrm{A}}^{\mathrm{n}+}\right)$  undergo a reduction reaction in the LFARFC by absorbing electrons to convert into low-valence redox ions $\left({\mathrm{A}}^{\left(\mathrm{n}-\mathrm{z}\right)+}\right)$. The VFB capacity recovery mechanism is based on the catalytic oxidation of formic acid at the anode during the discharge process to generate CO_2_ while releasing electrons. The high-valence VO_2_^+^ on the cathode side absorbs these electrons and is reduced to VO^2+^. As shown in Fig. 4b, the discharge curve of the LFARFC at 100 mA cm^−2^ is accompanied by the transition of the vanadium ion valence, and the cathode electrolyte color correspondingly changes from yellow (VO_2_^+^) to blue (VO^2+^). UV–Vis shows (Fig. 4c) that, after discharge, a clear VO^2+^ characteristic absorption peak appears at 765 nm in the electrolyte, based on which the average vanadium valence was calculated to be 4.02, indicating that approximately 98% of VO_2_^+^ is reduced to VO^2+^. The capacity recovery experiment was conducted at a constant current density of 200 mA cm^−2^, stopping after the 100th cycle, and the aforementioned repair strategy was adopted for treatment. The results indicate that its capacity can be restored to the highest level before the cycle (Fig. 4d). This LFARFC-based regeneration pathway avoids any continuous accumulation of foreign chemical impurities within the liquid phase, as the sole reaction by-product is gaseous carbon dioxide that naturally escapes from the open electrolyte tanks. Consequently, the pristine rheological properties and chemical composition of the bulk electrolyte are fully preserved over extended operation, allowing the post-recovery secondary degradation rate to remain highly stable (0.035% per cycle at 200 mA cm^−2^) [93]. Nicola et al. developed a capacity recovery system based on electrochemical regeneration principles. This system takes a standalone “regeneration cell” as the core and precisely corrects vanadium ion valence imbalance through electrochemical redox reactions. By coupling the reduction reaction of the positive electrolyte with the electrode-catalyzed OER, the increased average valence caused by side reactions such as HER can be forcibly reduced. A matching mathematical model was established to simulate in real time the dynamic response of the battery during the rebalancing process, thereby determining the optimal rebalancing endpoint to ensure that the imbalanced electrolyte (such as V^3.55+^ to V^3.65+^) is precisely restored to an ideal V^3.50+^ equilibrium state. Experiments show that the system achieves significant capacity recovery under the condition of an average CE of 81%, and the electrode performance is stable, providing a system-level solution for industrial-grade VFBs where adjusting the current density can shorten the recovery time, possessing good predictability and cost-effectiveness. This electrochemical rebalancing strategy uses only external electrical polarization, avoiding by-product accumulation and contamination. It preserves bulk electrolyte properties, resets the post-recovery degradation rate to its original baseline, and prevents accelerated capacity decay [94]. Pavel et al. proposed an in situ capacity recovery strategy based on chemical regeneration electrolyte circulation. Its core mechanism is to use an external electrolytic cell to electrochemically reduce excess VO_2_^+^ in the positive electrolyte to VO^2+^, coupling the spontaneous chemical reaction between a redox mediator and a cheap reducing agent (such as oxalic acid). In this mechanism, oxalic acid can efficiently reduce the oxidized mediator at room temperature while generating CO_2_ to be discharged from the system, thus precisely offsetting the increase in average oxidation state caused by side reactions such as HER without introducing impurity ions. Experimental results indicate that this strategy shows excellent stability and recovery efficiency in continuous VFBs cycling, and because OER at high potentials is avoided, electrode corrosion can be effectively mitigated. This study provides a low energy consumption, non-destructive system solution with industrialization potential for the long-term maintenance of RFBs capacity [95]. Electrode optimization to reduce imbalanced active species is an important research direction for the future. Roman et al. proposed an in situ method for initial capacity recovery. This strategy first performs partial electrochemical reduction of the positive electrolyte while performing OER on a RuO_2_/Ti electrode, subsequently followed by electrolyte mixing and recharging steps to restore battery performance to its initial state. The key to this method lies in adopting a composite electrode composed of RuO_2_ particles and a TiO_2_ oxide layer formed during the calcination process of titanium felt. Raman (Fig. 4e) and X-ray photoelectron spectroscopy (XPS) (Fig. 4f) characterization results confirm the existence of this composite structure. This electrode shows high catalytic activity for OER, can efficiently perform the reaction at low overpotentials, and exhibits excellent stability without significant degradation during the reaction process. Experiments indicate that this method can restore battery capacity completely to the initial level after 35 days of operation and 222 cycles, while the total energy efficiency (EE) of system only decreases by about 1.85% as a result [96]. Li et al. achieved effective recovery of system capacity by combining in line electrolysis technology on the basis of the reflux method, through reducing VO^2+^ accumulated in the positive electrolyte during long-term cycling. Experiments show that the capacity recovery effect is significant, and the VFB operates stably for 4680 cycles after eight in-line electrolysis treatments. This mainly benefits from the regulation of volume imbalance by the reflux method and the compensation of valence imbalance by in-line electrolysis. The researchers subsequently analyzed the composition of the positive electrolyte after the first cycle during the electrolysis process. As shown in Fig. 4g, as the reflux volume increases from 10 to 30%, the substance amounts of both VO^2+^ ions and total vanadium ions in the positive electrolyte rise. Correspondingly, the total vanadium ion amount in the negative electrolyte decreases with the increase of the positive reflux volume, a composition change trend is consistent with the recovery performance of the capacity retention rate after in-line electrolysis [97].

#### Other RFBs

**Fig. 5:**
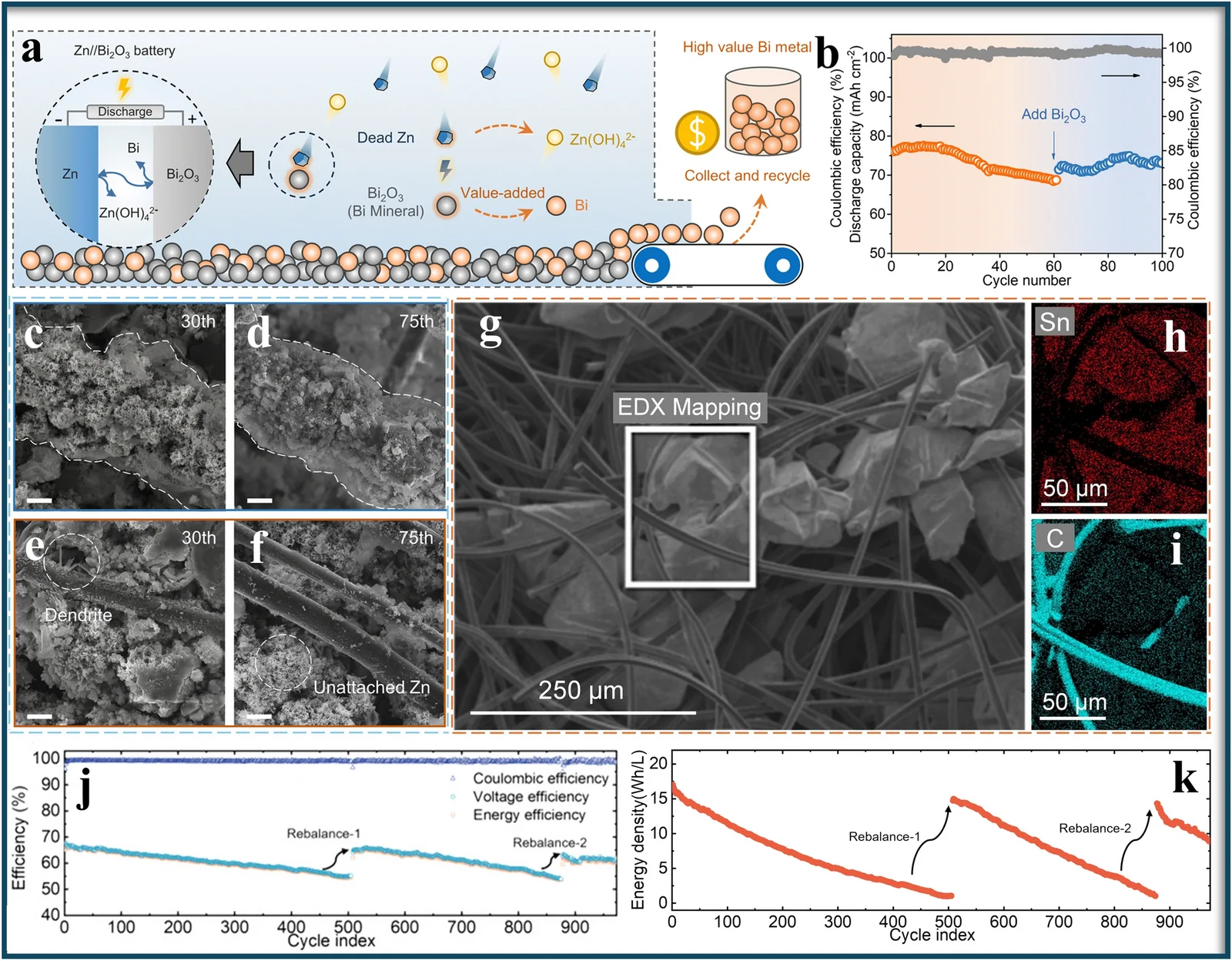
**a** Regeneration of dead zinc promoted by Zn–Bi_2_O_3_ chemistry in AZIFBs [101]. **b** Electrochemical performance of Zn/CF flow cell with Bi_2_O_3_ under capacity decay conditions [101]. SEM images of Zn deposition after several cycles at 80 mA cm^−2^ in AZIFBs with **c, d** and without **e, f** Bi_2_O_3_ [101]. Copyright 2024, Royal Society of Chemistry. **g-i** SEM imaging and EDX characterization of the Sn/Br RFBs anode [102]. Copyright 2019, Elsevier. Performance metrics of a tin–iron RFBs at 100 mA cm^−2^, including **j** CE, voltage efficiency (VE), EE, and **k** energy density [104]. Copyright 2025, Elsevier

In other RFB systems, precisely regulating and repairing valence imbalance triggered by side reactions such as HER through physical or electrochemical methods is also a key strategy for reversing capacity degradation and ensuring the long-term operational stability of the system. For zinc-based RFBs, in the repeated stripping and deposition process of the zinc negative electrode, zinc dendrites formed by nonuniform reactions may be flushed away from the electrode to the electrolyte during discharge to form “dead zinc,” the accumulation of which leads to irreversible battery capacity loss [98–100]. Wang et al. developed an electrochemically coupled Zn//Bi_2_O_3_ battery, composed of Bi_2_O_3_ and detached “dead zinc”. This system can react spontaneously during the discharge process, transforming inactive metallic zinc completely into Zn(OH)_4_^2−^ through Bi_2_O_3_-mediated electrochemical oxidation and re-releasing it into the anode electrolyte (Fig. 5a). This process effectively restores the concentration of Zn(OH)_4_^2−^ in the electrolyte, thereby achieving capacity recovery of zinc-based RFBs. To evaluate the regeneration ability of Bi_2_O_3_ for detached “dead zinc,” the research team tested Zn//CF RFBs without Bi_2_O_3_. The results show that the discharge capacity of this battery gradually decays starting from the 18th cycle, while after introducing Bi_2_O_3_, the capacity is significantly restored (Fig. 5b). SEM analysis indicates that in zinc-based RFBs containing Bi_2_O_3_, as cycling proceeds, the content of Bi and Zn-Bi alloy at the electrode interface gradually increases, and its inducing effect on zinc deposition is also enhanced accordingly, making the zinc tightly cover the fiber surface, thereby improving the cycle life of the battery (Fig. 5c, d). In contrast, zinc deposition in zinc-based RFBs without Bi_2_O_3_ presents a dispersed, loose granular form, with large chunks of deposits visible between fibers, and most fiber surfaces are not covered by zinc; this morphology instead promotes zinc dendrite growth and increases the zinc detachment rate (Fig. 5e, f). These results further confirm the key role of Bi_2_O_3_ in recovering “dead zinc” [101]. Zeng et al. reported a Sn/Br RFBs adopting a bromine-containing mixed electrolyte, possessing both high performance and low-cost characteristics and capable of achieving in situ capacity recovery. During the charging process, Br⁻ on the positive side is oxidized to Br_2_ or Br^3−^, while Sn^2+^ on the negative side gains electrons and is deposited on the negative electrode surface in the form of metallic tin; during discharge, the above process proceeds in reverse. This system drives tin ions that have migrated to the positive electrode back to the negative electrode and re-deposits them through reverse electrodeposition, thereby achieving in situ capacity recovery. Figure 5g–i shows the SEM image and corresponding EDX element distribution results of the negative electrode of the Sn/Br RFBs in a charged state. A significant Sn element signal can be detected on the carbon electrode surface. Combined with the fact that this deposition process is in a strongly acidic environment, it is further confirmed that the deposit on the negative electrode is metallic tin. Cycle tests show that after this battery undergo more than 250 charge–discharge cycles at 120 mA cm^−2^ and 15 °C, the discharge capacity shows a gradual decay trend; after in situ recovery treatment, its capacity could be restored to the initial level [102]. In Fe–Cr RFBs, electrolyte imbalance and capacity degradation caused by HER reaction are particularly significant. Zeng et al. proposed and verified an in situ repair mechanism based on a hydrogen-iron ion-rebalancing battery, using the hydrogen generated at the negative electrode as a reducing agent to reduce excess Fe^3+^ in the positive electrode to Fe^2+^ through electrons provided by the oxidation reaction in the rebalancing battery, thereby restoring the chemical balance of the electrolyte and battery capacity. Research indicates that adopting an interdigitated flow field design can significantly enhance the mass transfer efficiency under low-concentration hydrogen, making the utilization rate of hydrogen close to 100% when the concentration is lower than 5%, and can achieve continuous stable rebalancing at 60 mA cm^−2^. The cost of this device only accounts for about 1% of the total system cost, indicating its good economic and practical value in maintaining long-life battery operation and improving system safety [103]. Xue et al., while adopting the hydrogen-Fe^3+^ rebalancing method to alleviate Fe^3+^ accumulation, also introduced a sulfate–chloride mixed acid as the supporting electrolyte, further improving system stability and inhibiting ion transmembrane penetration. Subsequently, long-term cycle tests with the aforementioned mixed acid at 25 °C further verify the effectiveness of this rebalancing strategy. After 500 cycles, due to serious SOC imbalance, the battery capacity significantly drops to 1.03 Wh L^−1^. Strikingly, after only two rebalancing cycles, the energy density was restored from 1.03 to 14.98 Wh L^−1^ (Fig. 5j, k). Subsequent continuous application of rebalancing cycles allows the energy density to be stably maintained at approximately 14.60 Wh L^−1^, reflecting the continuous recovery capability of this method. Furthermore, the CE throughout the cycle is always higher than 98%, and after each rebalancing, both VE and EE are significantly improved, fully proving the reliability and practicality of the hydrogen-mediated reduction strategy. Besides, this native hydrogen iron rebalancing framework establishes a completely closed-loop material cycle that utilizes the self-generated hydrogen by-product to restore the state-of-charge balance. Because no external chemical species are added to the system, the electrolyte properties remain perfectly intact, effectively stabilizing the post-recovery secondary degradation rate [104].

AORFBs, as an emerging electrochemical energy storage system, also face unavoidable capacity degradation problems during long-term cycling [105]. Therefore, developing effective capacity recovery methods is of great significance for their practical application. Guiheneuf et al. used 2, 3-dihydroxylated anthraquinone as the active species in AORFBs, which has extremely high solubility in alkaline electrolytes and high stability, not easily decomposing to cause capacity degradation. Research indicates that in the first 180 cycles, the reversible capacity degradation rate of the battery is 0.45% per day, EE is 84.3%, and the maximum power density is 189 mW cm^−2^. In subsequent cycle tests lasting up to 3000 times, the battery still shows good stability, with the capacity degradation rate of 0.8% per day within the first 2400 cycles and the maximum power density of 170 mW cm^−2^. Importantly, this system can achieve the recovery of initial capacity through deep discharge, proving reversible capacity degradation, thus providing an important material basis for the capacity recovery of AORFBs [106]. Chinmaya et al. synthesized an anthrarufin-based polymer—1, 5-dihydroxy-9,10-dioxo-9,10-dihydroanthracene-2,6-disulfonic acid (DSAN)—as the active species, which has extremely high solubility and reversibility in alkaline electrolytes. To solve the problem of significant capacity degradation caused by SOC mismatch between positive and negative electrodes due to HER of the negative active species (DSAN^2⁻^), this work proposed an in situ electrolysis rebalancing recovery strategy. This strategy induces OER on the positive side to consume excess oxidized active species by increasing the charge cut-off voltage, while reducing DSAN back to DSAN^2−^ on the negative side, thereby correcting the SOC deviation and achieving a reset of system capacity. Experiments show that this method can restore a battery that has lost 80% of its capacity to near-initial capacity levels multiple times. If D-fructose is further added to the negative electrolyte to synergistically inhibit HER, the capacity retention rate of the battery after 100 cycles can reach 96.5%, significantly improving the cycle life and operational stability of AORFBs. Significantly, this in situ high-voltage reset protocol rebalances the system through purely electrical control and native electrochemical pathways, having no adverse effect on the entire system. Furthermore, the secondary capacity decay rate of this recovery strategy is extremely low, with an average decay rate of only 0.035% per cycle [107].

In summary, precisely regulating the valence of active species is a key technology for coping with capacity degradation during long-term cycling of RFBs. Through strategies such as chemical reduction (e.g., oxalic acid, formic acid), in situ/online electrolysis, reverse electrodeposition, and heterojunction interface induction, researchers have successfully achieved reactivation of inactivated species and significant recovery of capacity in VFBs, zinc-based systems, Zn-Br RFBs, and AORFBs. From the correction of over-oxidized states in VFBs, to “dead zinc” regeneration based on Bi_2_O_3_ mediation in zinc-based RFBs, to SOC rebalancing induced by OER in organic systems, these methods not only prove the reversibility of valence imbalance but also significantly extend the actual cycle life of RFBs. Unlike traditional chemical additives, oxalic acid and formic acid function as small-molecule organic remedies whose ultimate oxidation by-products are predominantly volatile gaseous molecules such as carbon dioxide. Thermodynamics dictate that these organic species generate no irreversible solid inorganic precipitates within the supporting bulk solution or across the active electrode interfaces, thereby eliminating the traditional root causes of hard physical pore blockages. Nevertheless, under the rigorous operating conditions of tens of thousands of continuous cycles, the localized dehydrogenation or oxidation cascades of formic or oxalic acid on highly porous carbon felt matrices can potentially generate strongly bound oxygenated intermediates. These transient chemical species tend to occupy the highly active defect sites of carbonaceous fibers, causing a pronounced escalation in interfacial charge transfer resistance, which subsequently drives the macroscopic performance degradation of RFBs. Besides, this strategy still faces certain limitations in practical application, mainly manifested as the recovery process being difficult to operate efficiently at current densities exceeding 200 mA cm^−2^, and in most cases, complete recovery of the lost capacity cannot be achieved. In the future, artificial intelligence (AI) technology can be used to screen or design from scratch new molecular or material reducing agents that possess both high reaction selectivity and excellent stability to overcome problems such as insufficient reaction kinetics of traditional reducing agents, thereby promoting new breakthroughs in current density and capacity recovery rate.

### Electrode Active Site Repair and Transport Optimization

**Fig. 6:**
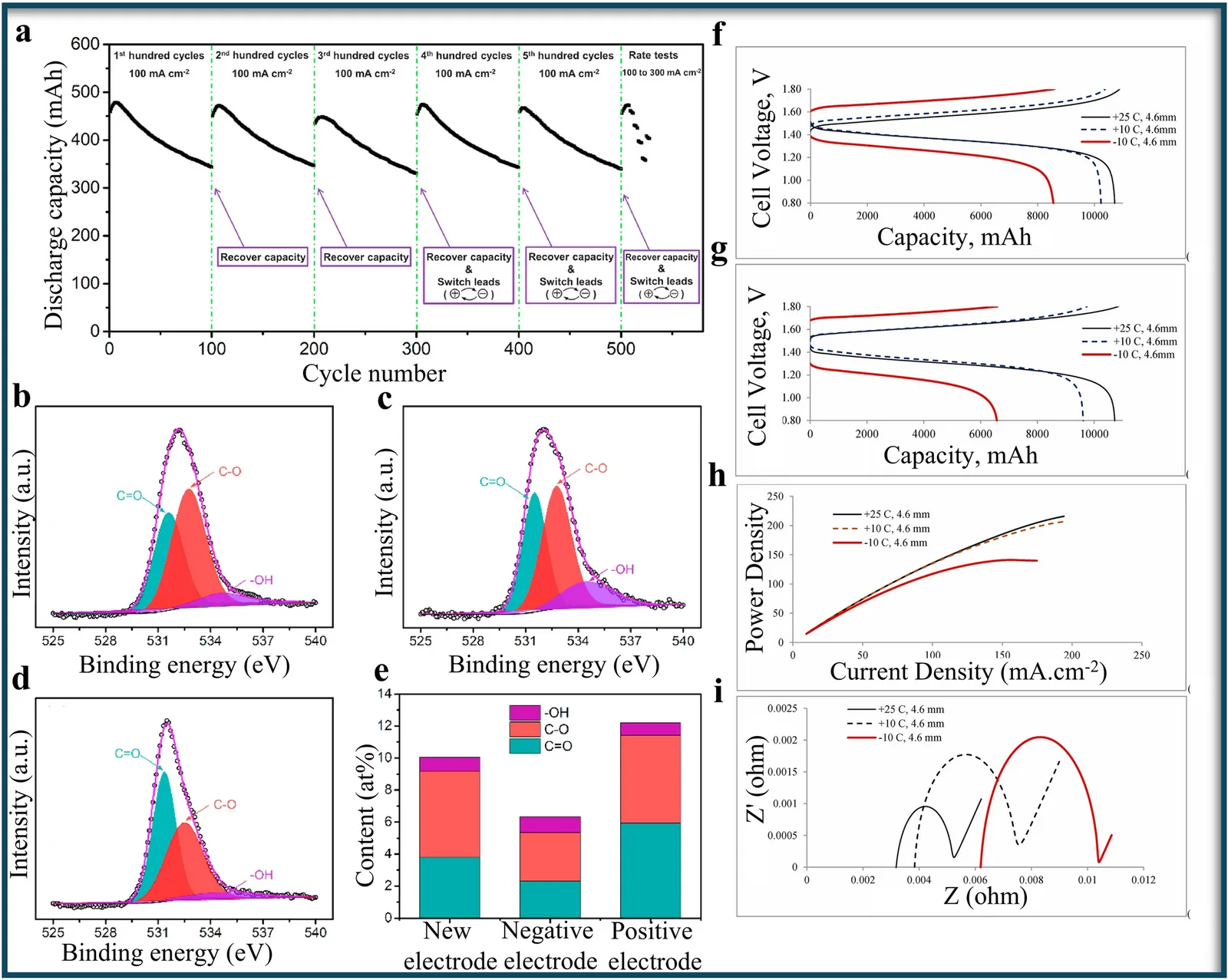
**a** Discharge capacity maintenance over the cycling period [110]. XPS results in the O1s from different electrodes:** b** new electrode, **c** negative electrode after cycle operation, and **d** positive electrode after cycle operation. **e** Distribution of types of oxygen-functional groups for different electrodes [110]. Copyright 2020, Elsevier. Polarization characteristics under **f** low circulation and **g** high-rate (80 mA cm^−2^, SF_50_ = 9.9) conditions [111]. **h, i** Comparative analysis of power densities and cell impedances [111]. Copyright 2021, Elsevier

Regenerating electrode active sites is key to improving RFBs kinetic performance. By repairing oxygen-functional groups on the electrode surface or clearing pollutants accumulated during long-term cycling, charge transfer impedance and activation overpotential can be effectively reduced. This polarization inhibition can significantly delay the time for the battery to reach the cut-off voltage during charge–discharge, thereby achieving efficient recovery of RFBs available discharge capacity without changing the electrolyte composition [108, 109]. Wei et al. almost completely restored the EE decay caused by electrode degradation by swapping the positive and negative electrodes of the RFBs without dismantling the battery. This is because the electrodes that originally undergo surface oxidation or functional group inactivation in the positive electrode environment can clear surface oxide films or pollutants through reduction after switching to the negative electrode environment, restoring their electrochemical activity. Battery performance tests find that after regeneration by this method, battery performance recoveries significantly, maintaining a capacity of 473 mAh (Fig. 6a) and an EE of 90.8% after 500 cycles, basically maintaining the optimal performance level at the beginning of the cycle (478 mAh, 91.0%). The researchers then performed XPS analysis, the O 1 s core-level spectra of the initial electrode, negative electrode after cycle operation, and positive electrode after cycle operation are shown in Fig. 6b-d, and the distribution of types of oxygen-functional groups is summarized in Fig. 6e. Results show that the binding energy peaks at 534.7, 532.5, and 531.5 eV correspond to –OH, C–O, and C=O functional groups, respectively. The total content of oxygen-functional groups increases in the following order: negative electrode (6.3%) < pristine electrode (10.1%) < positive electrode (12.2%). Specifically, the content of C=O groups in the positive electrode relatively increases, while the content of both C=O and C–O groups in the negative electrode decreases. These changes further confirm that the composition and distribution of oxygen-functional groups have a significant influence on VFBs performance. This purely physical approach avoids the impact of adverse chemical reactions; however, since the electrolyte is not replaced, the secondary capacity decay rate may be slightly higher [110]. Under extreme conditions such as low-temperature environments, increased electrolyte viscosity and decreased conductivity will trigger serious ohmic polarization and mass transfer polarization. By measures such as optimizing the electrode compression ratio to reduce internal resistance, overpotentials can be significantly reduced and the time for the battery to reach cut-off voltage can be delayed, thereby effectively recovering the apparent capacity lost due to polarization limitations. Praphulla et al., while optimizing the electrode compression ratio, also adopted thinner electrodes to further reduce resistance. Polarization curves show that an electrode with a thickness of 4.6 mm and a compression ratio of 35% exhibits significantly improved discharge capacity at current densities of 60 and 80 mA cm^−2^. Even at a low temperature of − 10 °C, this thin electrode could still maintain about 60% of the initial discharge capacity. Simultaneously, the peak power density of the battery increases by approximately 15% (Fig. 6f–h). Electrochemical impedance spectroscopy analysis indicates that at different temperatures, using thinner electrodes can reduce ohmic resistance by 15%–20%; especially at − 10 °C, the ohmic resistance decrease exceeded 50% (Fig. 6i). These improvements together promote the further enhancement of overall battery performance [111].

In summary, periodic electrode swapping enables in situ regeneration of active sites, while electrode structure optimization effectively suppresses polarization. The above strategies provide a capacity recovery pathway that does not rely on adjusting electrolyte composition and show significant recovery efficiency under extreme working conditions. However, existing physical repair means mainly target capacity degradation at the electrochemical kinetics level, and it is difficult to fundamentally solve the charge imbalance problem caused by transmembrane migration of active species or deep side reactions. Besides, although swapping positive and negative electrodes offers a low-cost pathway for regenerating active sites, scaling this approach to industrial stacks introduces notable engineering complexities. In multi-cell configurations, residual electrolyte trapped within manifolds and porous electrodes may trigger transient self-discharge or parasitic side reactions during the swapping procedure. Additionally, the requirement for sophisticated piping and valve manifolds to maintain seamless flow reversal without air ingress can substantially elevate the initial capital expenditure. Therefore, such strategies are most effectively deployed in symmetric systems or those utilizing high tolerance membranes to minimize the risk of irreversible ion mixing. Future research needs to further explore the synergistic mechanism between physical kinetic recovery and chemical component rebalancing to achieve more comprehensive capacity recovery of RFBs.

### Model-Driven Collaborative Recovery Strategies

Model-algorithm-driven collaborative recovery strategies mark a transition from passive maintenance to system-level precise scheduling. This strategy, by integrating multi-physics field data, achieves deep coordination of electrolyte volume rebalancing, charge valence correction, electrode degradation prediction, and HER side reaction inhibition [112, 113]. Relying on digital twins or deep learning algorithms, this strategy can decouple the health state of RFBs in real time, accurately predicting the optimal intervention timing and repair effect for capacity recovery, thereby seeking the optimal solution between physical fluid distribution and chemical redox balance, ensuring that the battery always maintains optimal capacity utilization and operational stability throughout its full life cycle.

**Fig. 7:**
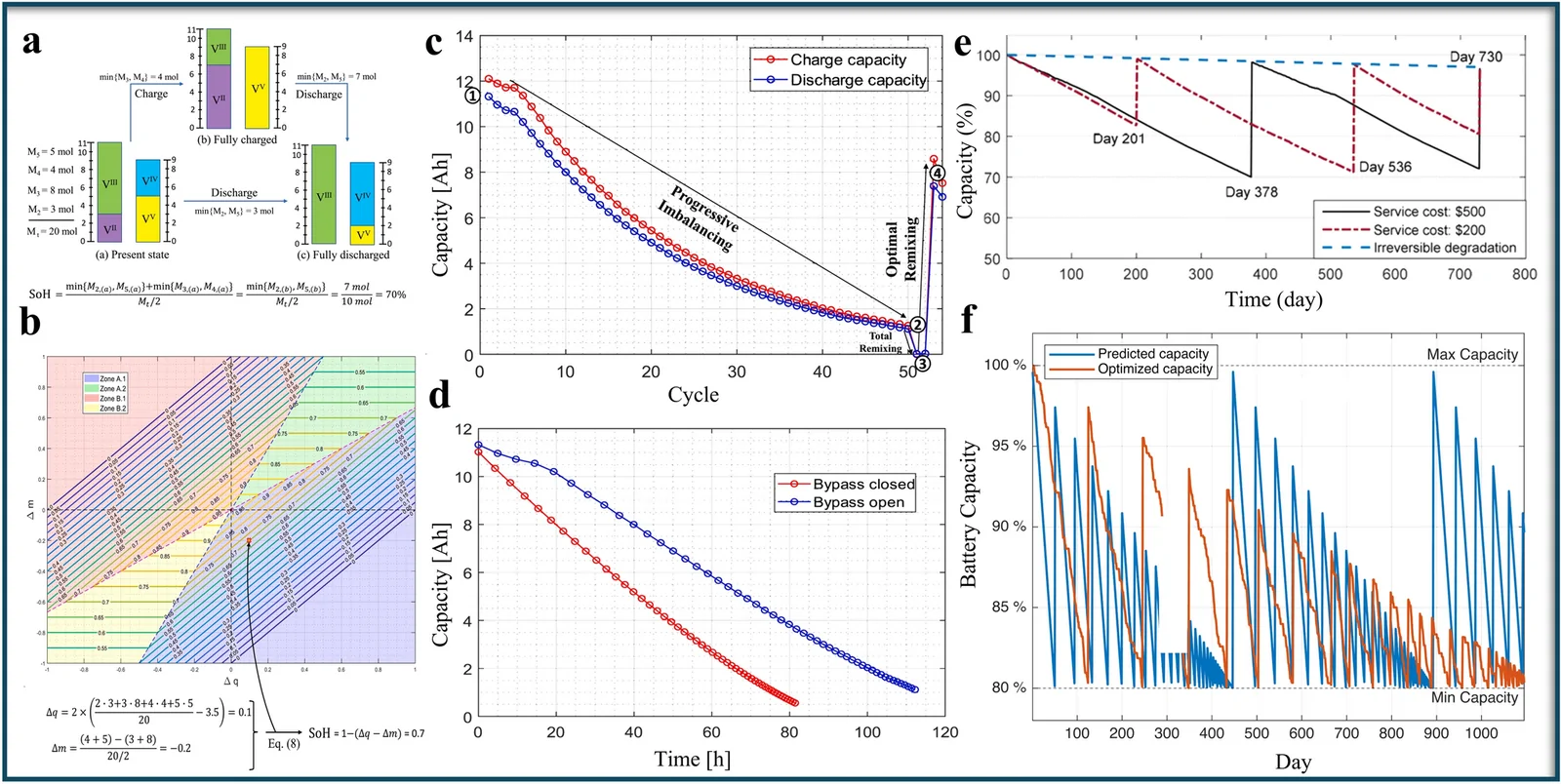
**a** Conceptual illustration of the generalized SoH [114]. **b** SoH contour curves on the Δm-Δq imbalance map [114]. **c** Capacity fading characteristics induced by imbalance and the recovery efficiency of different remixing methods [114]. **d** Comparison of the capacity evolution in the first 50 cycles of Test 1 (bypass closed) and Test 2 (bypass open) [114]. Copyright 2024, Elsevier. **e** Service cost-dependent optimization of VFBs restoration scheduling [116]. **f** Comparison of capacity decay versus maintenance-enhanced stability over a three-year operational lifespan [116]. Copyright 2023, Elsevier

Thomas et al. designed a new optimized partial remixing procedure to recover VFBs capacity loss caused by electrolyte imbalance. The researchers used a general expression (Eq. 2) to calculate the state of health (SoH) of VFBs under any degree of Faradaic imbalance and stoichiometric imbalance, where ${M}_{i}$ is the number of moles of

2 $${\mathrm{SoH}} = \frac{{\min \{ M_{2} ,M_{5} \} + \min \{ M_{3} ,M_{4} \} }}{{M_{t} /2}}$$

vanadium ions with oxidation state +i and ${M}_{t}$ is the total number of vanadium moles in the system: ${M}_{t}={M}_{2}+{M}_{3}+{M}_{4}+{M}_{5}$. Figure 7a shows that in the case of positive and negative imbalance, only about 7 mol of vanadium ions participate in the reaction when the battery runs from full charge to full discharge, while in an equilibrium state, the ions participating in the reaction can reach 10 mol. This significant reduction in reaction ions directly leads to battery capacity degradation. To visually demonstrate the influence of the stoichiometric imbalance index (∆m) and the Faradaic imbalance index (∆q), the researchers first derived Eq. 3, then calculated the average oxidation state (AOS) using Eq. 4, and finally obtained Eq. 5:

3 $$\Delta m = \frac{{\left( {M_{4} + M_{5} } \right) - \left( {M_{2} + M_{3} } \right)}}{{M_{t} /2}}$$

4 $${\mathrm{AOS}} = 2\frac{{M_{2} }}{{M_{t} }} + 3\frac{{M_{3} }}{{M_{t} }} + 4\frac{{M_{4} }}{{M_{t} }} + 5\frac{{M_{5} }}{{M_{t} }}$$

5 $$\Delta q = 2\left( {{\mathrm{AOS}} - 3.5} \right)$$

Figure 7b shows SoH contour curves based on ∆m and ∆q, further confirming the electrolyte imbalance of VFBs. The researchers subsequently simulated the effect of physical recovery means. Figure 7c shows a profile of the battery discharge capacity during cycles (plunging from 11.2 to 1.3 Ah) after installing a bypass tube device, indicating that physical rebalancing means have a certain promotional effect on capacity recovery. Figure 7d indicates that the bypass tube can effectively delay (rather than eliminate) the rate of capacity degradation. The researchers finally found, by transferring the calculated electrolyte volume from the negative side to the positive side, that this strategy can significantly alleviate the capacity degradation caused by oxidative imbalance. In short, this procedure can serve as an effective means for predicting capacity recovery [114]. Mehdi et al. proposed a two-step mixed-integer linear programming algorithm to optimize the capacity recovery process of VFBs. This algorithm first determines the reasonable range for the optimal number of maintenance events and then jointly optimizes the specific time and frequency of each maintenance event. Figure 7e shows the optimal maintenance schedule in two cases: reversible and irreversible capacity degradation. The results show that, influenced by factors such as energy price fluctuations, the interval days between each maintenance are not fixed. This algorithm automatically generates maintenance plans based on health state prediction, which can effectively avoid energy loss and additional labor costs caused by unnecessary electrolyte mixing operations [115]. Diana et al. used Convex Hulls to linearize the RFBs capacity degradation problem. In addition, this optimization program also calculated the progressive capacity degradation caused by unintended side reactions (Eq. 6), where $\widehat{{R_{{{\mathrm{fade}}}} }}$ is the total capacity fade rate, $\widehat{{r_{{{\mathrm{CR}}}} }}$ is crossover fade rate and $\widehat{{r_{{{\mathrm{ED}}}} }}$ is electrolyte decay rate due to oxidative imbalance.

6 $$\widehat{{R_{{{\mathrm{fade}}}} }} = \widehat{{r_{{{\mathrm{CR}}}} }} + \widehat{{r_{{{\mathrm{ED}}}} }}$$

The researchers used the aforementioned algorithm program to precisely predict the optimal capacity recovery time nodes. Figure 7f shows the changes in the available capacity ratio of a typical household wind power energy storage scenario during three years of continuous operation. The figure compares the available capacity ratio optimized by this program with the ideal capacity ratio predicted based on fixed daily cycle number assumptions. The results indicate that traditional constant cycle assumptions often misjudge the actual capacity degradation trajectory of long-duration energy storage systems. The optimization algorithm proposed in this work can dynamically describe the capacity evolution process based on real-time power fluctuations and market arbitrage demand, thereby achieving precise prediction of capacity recovery nodes [116]. Kara et al. used a levelized cost of storage (LCOS) model to conduct a techno-economic assessment of repair strategies for asymmetric RFBs. This model combines linear capacity degradation caused by electrolyte penetration and chemical degradation with economic indicators, dynamically tracking the energy and cost components throughout the battery life cycle by setting a capacity lower limit to trigger corresponding maintenance measures (such as electrolyte rebalancing or active species replacement). The assessment focuses on comparing the economic differences between finite-life (such as organic molecules) and infinite-life active species under different repair pathways. The results indicate that capacity recovery capability should be regarded as a core criterion for battery design. For asymmetric systems, if they are to compete with VFBs, they must ensure that active species have extremely low degradation rates or achieve extremely low-cost capacity recovery while reducing initial investment. This model provides a quantitative decision-making framework for capacity recovery strategies, and by simulating the impact of different repair plans on LCOS, it can assist in determining the optimal maintenance frequency and timing, and clearly reveal the economic trade-off between initial material cost and long-term maintenance expenditure [117].

In summary, model-driven collaborative recovery strategies, by integrating multi-algorithms such as AOS and SoH characterization, mixed-integer linear programming, and LCOS analysis, have achieved a transformation of the RFBs operational maintenance mode from “passive response” to “systematic precise scheduling,” providing a quantitative decision-making basis for optimizing capacity recovery nodes, maintenance frequency, and full-life-cycle economics. However, under actual complex operating conditions, such idealized mathematical models often produce prediction deviations due to factors such as nonlinear electrolyte degradation, extreme working condition fluctuations, and multi-field coupled side reactions. Therefore, there is an urgent need to develop intelligent regulation systems that fuse high-precision models with dynamic experimental verification, continuously correcting algorithm parameters through real-time in situ monitoring data, and building a collaborative recovery system integrating prediction and experimental closed- loop.

### Stack-Level Capacity Maintenance and Practical Recovery Management

Transitioning from localized single-cell degradation boundaries to multi-kilowatt commercial stacks introduces complex macroscale stress vectors that accelerate system capacity loss and operational imbalance. At the stack scale, shunt currents continuously flowing through the shared conductive electrolyte distribution manifolds trigger chronic parasitic faradaic reactions, directly causing rapid divergence in state of charge among edge cells. This spatial state-of-charge nonuniformity is further amplified by nonuniform hydraulic flow distribution across poorly optimized flow frames, where peripheral cells experience mass transport limitations and high transient charging overpotentials. Concurrently, severe thermal fluctuations and localized heat accumulation accelerate water evaporation via the storage reservoir exhaust, altering the supporting acid concentration matrix and triggering chemical precipitation vectors. Managing these stack-scale operational failures requires comprehensive engineering recovery frameworks that map real-time multi-physics fluctuations to actionable control loops before permanent system shutdown events are triggered.

To effectively mitigate these macroscale decay pathways, advanced diagnostic systems must be deployed to capture real-time indicators of stack degradation. For instance, the onset of shunt currents is typically manifested by interfacial terminal-voltage divergence among individual cells and localized high-temperature thermal anomalies along the distribution manifolds. Similarly, continuous water evaporation can be accurately detected by monitoring the asymmetry of the bulk liquid level and the progressive increase in density within the acid reservoirs. When severe thermal fluctuations occur, they generate pronounced spatial temperature gradients across the stack assembly, which can be tracked alongside accelerated active material precipitation in localized hot zones. Finally, the nonuniformity of internal electrochemical states can be diagnosed through single-cell voltage divergence during continuous cycling and premature capacity depletion, indicating that specific cells are operating outside their optimal thermodynamic windows.

Addressing these quantified indicators requires a matrix of targeted engineering recovery actions that balance materials sustainability with automated plant control. Manifold shunt currents can be suppressed by optimizing common electrolyte channel lengths to maximize ionic resistance and by deploying periodic system wide open circuit rest cycles to dissipate accumulated residual potentials. To counteract operational water evaporation, systems must incorporate automated deionized water injection loops paired with closed-loop gas condensing circuits at the reservoir exhausts. Furthermore, severe thermal fluctuations are systematically managed by interfacing high thermal conductivity heat exchangers with proactive smart heating or cooling jacket networks to homogenize the stack internal temperature profile. To compress cell-level state-of-charge divergence, operators can deploy automated cell-level balance circuits or execute periodic complete fluidic remixing operations to reset the global stoichiometric inventory.

Despite the efficacy of these active restoration protocols, practical implementation within large-scale commercial facilities remains bounded by inherent engineering limitations. Optimizing manifold geometry to lower shunt currents inevitably induces a minor but permanent parasitic energy efficiency deduction due to increased flow resistance, which cannot be completely bypassed physically. Automated water replenishment systems require high sensitivity fluidic level instrumentation that elevates initial capital costs and increases total system weight penalties. Moreover, deploying high thermal conductivity heat exchangers and smart thermal jackets demands high initial capital expenditure and significantly complicates the computational control framework of the system. Finally, while individual cell balancing circuits and complete fluidic remixing successfully eliminate state-of-charge divergence, they are inherently time-consuming and introduce substantial complexity into the overall balance of plant electrical hardware controls (Table 1). The above limitations also represent key research directions for future stack-scale RFBs.

**Table 1:** Stack-level capacity degradation diagnostic indicators recovery actions and remaining limitations

Stack-level degradation sources	Practical diagnostic indicators	Engineering recovery actions	Remaining limitations
Shunt currents in manifolds	Interfacial voltage divergence and localized thermal anomalies	Optimizing channel lengths and deploying periodic open circuit rest cycles	Induces a permanent parasitic EE deduction within the system
Water evaporation via exhaust	Bulk liquid-level asymmetry and reservoir density increase	Implementing automated water injection and closed gas condensing circuits	Requires sensitive instrumentation increasing system weight penalties
Severe thermal fluctuations	Spatial temperature gradients and accelerated hot zone precipitation	Interfacing high conductivity heat exchangers with smart jackets	Demands high capital investments complicating system computational frameworks
Nonuniform SOC distribution	Single-cell voltage divergence and premature capacity depletion	Deploying cell-level balance circuits or executing fluidic remixing	Prolongs system downtime complicating balance of plant hardware controls

Active recovery protocols can effectively restore capacity in RFBs, but inevitably introduce parasitic energy consumption and operational downtime in MW-scale commercial systems. To objectively assess their practical viability, the community should establish standardized techno-economic analysis (TEA) frameworks that integrate real-time system energy efficiency loss, cumulative fiscal costs from scheduled shutdowns, and long-term levelized cost of storage. Such frameworks must balance the capital expenditure of auxiliary rebalancing equipment against the financial benefits of extended system lifetime, thereby avoiding net negative energy returns over multi-decade grid deployment. In parallel, TEA should be rigorously coupled with life-cycle assessment (LCA) to monitor the long-term environmental and material footprints of active recovery cycles. Invasive chemical resetting or sustained high-voltage in-line electrolysis can alter the baseline degradation trajectories of stack components, shifting the global carbon footprint and material recyclability of the storage facility. A standardized LCA protocol enables operators to track how localized chemical additives or periodic cleaning workflows affect stack sustainability over thousands of consecutive MWh [118]. Developing these unified analytical tools will help transition the field from empirical laboratory demonstrations to a mature industrial sector where environmental sustainability and operating income are dynamically co-optimized.

## Summary and Outlook

### Concluding Remarks

In summary, mitigating capacity degradation in RFBs is a critical prerequisite for ensuring the economic viability and operational stability of long-duration energy storage systems. This review has systematically elucidated multi-dimensional failure mechanisms, covering physical electrolyte imbalances driven by transmembrane migration, chemical instabilities such as active species inactivation, and interfacial kinetic degradation triggered by parasitic gas evolution or electrode passivation. Current capacity recovery strategies: ranging from source-level hydraulic rebalancing via high-selectivity membranes and electrode active-site repair with transport optimization to precise electrochemical valence regulation and model-driven intelligent scheduling—have demonstrated significant efficacy, with some advanced methods achieving near 100% capacity recovery (Table 2). By comparing various strategies (Table 3), it is found that physical restoration strategies, such as periodic electrode swapping or structural optimization, are cost-effective due to their independence from expensive chemical reagents; however, they encounter significant engineering hurdles in industrial-scale high-power stacks, including piping complexity and required system downtime. It is worth noting that in RFB systems, the cost of the electrolyte typically accounts for 50% or even more of the total cost. Therefore, valence regulation methods such as chemical reduction and online electrolysis can significantly improve capacity recovery and substantially reduce the cost of electrolyte replacement. However, kinetic limitations at high current densities often lead to increased parasitic energy losses and system complexity, which also contribute to a certain degree of cost escalation. In comparison, integrating specialized auxiliary rebalancing devices, such as hydrogen-iron rebalancing cells, requires a hardware investment of only approximately 1% of the total system cost, offering exceptional industrial potential. Ultimately, within a levelized cost of storage framework, utilizing digital twins and model-driven intelligent scheduling for the precise prediction of optimal maintenance intervals can minimize energy dissipation and maximize the system full life cycle, serving as the primary route to achieving economic competitiveness for RFBs in the large-scale long-duration energy storage market. Inevitably, the capacity recovery strategies discussed above affect the electrochemical performances of RFBs. For instance, physical restoration and in situ interfacial methods eliminate kinetic overpotentials and restore EE to its pristine baseline, whereas periodic electrode swapping may introduce localized parasitic self-discharge in industrial multi-cell stacks. In contrast, advanced closed-loop chemical pathways (e.g., gas-evolving reduction or hydrogen-mediated rebalancing) preserve the electrolyte’s native chemical integrity, ensuring stable ionic conductivity and viscosity over long periods. Even when online electrolysis incurs a minor energy penalty, low overpotential composite catalytic electrodes limit the total EE loss to a negligible 2%, which is vastly outweighed by the long-term LCOS benefits of permanent capacity restoration. However, as the industry moves toward high-power and large-scale industrial deployment, the field still faces deep-seated scientific and technical challenges that necessitate further breakthroughs.

**Table 2:** Typical RFBs capacity recovery strategies, operational parameters, and capacity recovery rates

Recovery strategies	RFB types	Initial total capacity (mAh)/areal capacity (mAh cm^−2^)/volumetric capacity (Ah L^−1^)	Current density (mA cm^−2^)	Total capacity after recovery (mAh)/areal capacity (mAh cm^−2^)/volumetric capacity (Ah L^−1^)	Capacity recovery rate (%)	Cycle numbers after recovery	References
Electrolyte volume rebalancing	Ti/Ce RFBs	101.12 mAh	50	101.12 mAh	100	3721	[85]
	VFBs	23.5 Ah L^−1^ (0.05 L)	100	23 Ah L^−1^	98	–	[87]
Precise regulation of active species valence	VFBs	481.0 mAh	200	469.6 mAh	97.6	–	[93]
	VFBs	1400 mAh	160	123 mAh	88.2	4680	[97]
	AZIFB	99.5 mAh cm^−2^ (4 cm^2^)	160	98.5 mAh cm^−2^	99	–	[101]
	Sn/Br RFBs	214.4 mAh	120	197.2 mAh	83.6	–	[102]
	Fe/Cr RFBs	630 mAh	60	592 mAh	94	–	[103]
	AORFBs	37.5 Ah L^−1^ (0.11 L)	100	37.5 Ah L^−1^	100	3000	[106]
	AORFBs	5.9 Ah L^−1^ (0.03 L)	20	5.9 Ah L^−1^	100	–	[107]
Electrode active-site repair and transport optimization	VFBs	478 mAh	100	472 mAh	98.7	500	[110]

**Table 3:** Representative techniques, key advantages, and limitations of various recovery strategies

Recovery strategies	Corresponding degradation mechanisms	Typical approaches	Core advantages	Major limitations
Electrolyte volume rebalancing	Volume imbalance of positive and negative electrolytes	Highly selective membrane	Effectively obstructs parasitic mass crossover via size exclusion	Increases stack internal resistance and reduces voltage efficiency
Precise regulation of active species valence	Chemical instability of active materials	Online electrochemical electrolysis and chemical reduction	Completely eradicates faradaic state-of-charge deviations	Incurs efficiency penalties and by-product accumulation
Electrode active-site repair and transport optimization	Electrode degradation and gas evolution	Electrode interface optimization and periodic structural electrode polarity swapping	Eliminates kinetic charge transfer resistance overpotentials	Introduces severe fluidic sealing and mechanical complexities
Model-driven collaborative recovery strategies	-	Deep neural network predictive algorithms and real-time digital twin	Optimizes maintenance intervals dynamically under fluctuating grid profiles	Requires high computational investments and accurate training data

Furthermore, it is essential to emphasize that a strict distinction exists between reversible capacity fading and irreversible capacity loss. The overwhelming majority of contemporary capacity recovery strategies are designed exclusively to counteract reversible capacity fading, such as bulk electrolyte volume redistribution or SOC valence drift. These transient imbalances can be completely rectified through routine automated physical remixing or operando online electrochemical polarization, without altering the intrinsic material structure. In contrast, conventional engineering adjustments remain fundamentally ineffective against irreversible capacity decay pathways, which are rooted in permanent chemical alterations, including the molecular decomposition of organic active substances and severe carbon corrosion of the substrate. Therefore, developing recovery strategies applicable to irreversible capacity fading is also a major research direction in the field of RFBs.

### Challenges and Future Perspectives

**Fig. 8:**
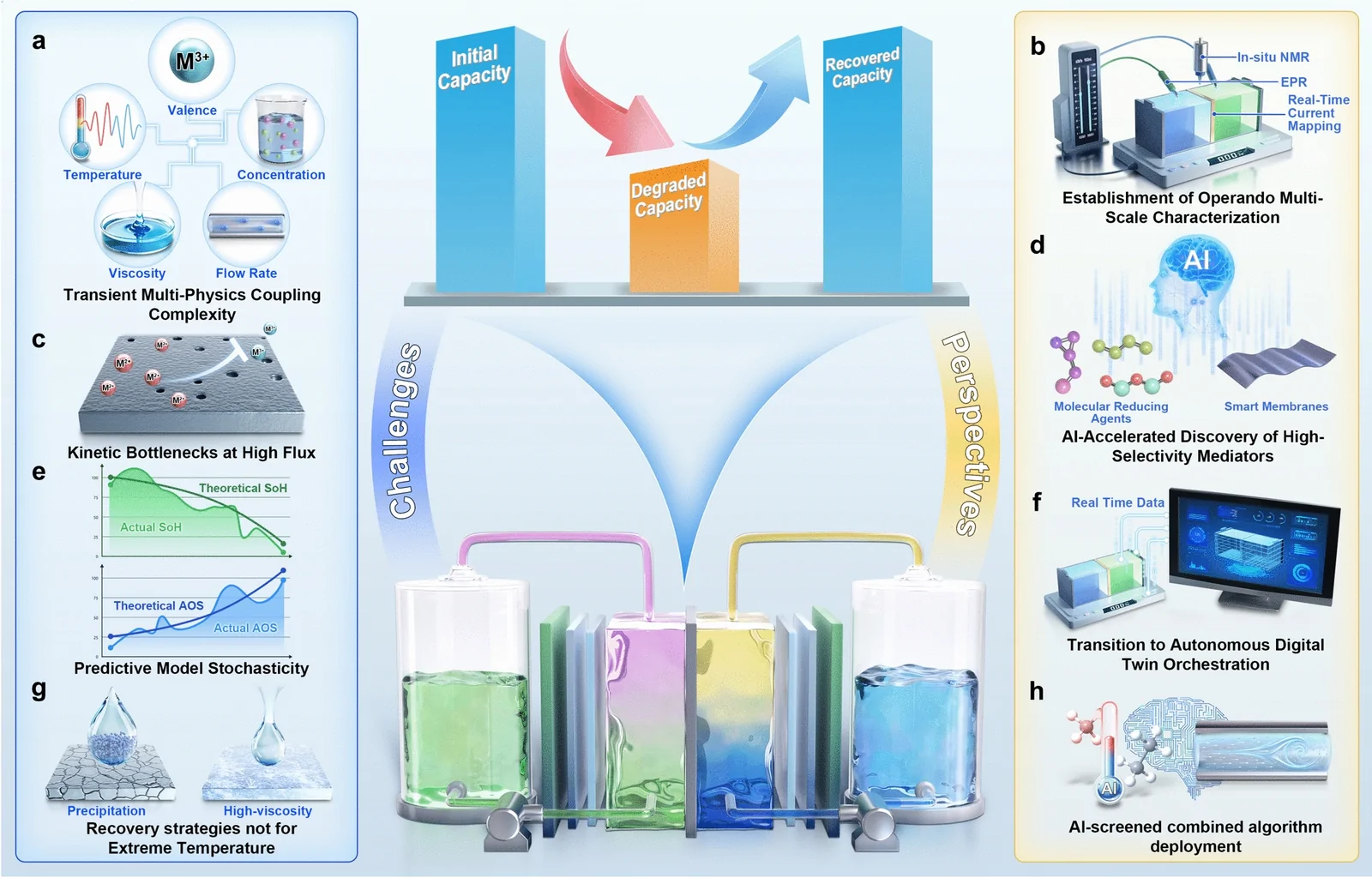
**a** Transient multi-physics coupling complexity. **b** Establishment of operando multi-scale characterization. **c** Kinetic bottlenecks at high flux. **d** AI-accelerated discovery of high-selectivity mediators. **e** Predictive model stochasticity. **f** Transition to autonomous digital-twin orchestration. **g** Unsuitability of recovery strategies at extreme temperatures. **h** AI-screened combined algorithm deployment

While individual capacity recovery technologies have demonstrated feasibility in localized laboratory settings, the RFB community still faces deep-seated, systemic barriers when transitioning from descriptive postmortem diagnostics to actionable, lifetime-extending interventions. Based on the comprehensive cross-coupling analysis of multi-dimensional degradation roots presented in this review, we summarize the following specific, exclusive research gaps and actionable future directions:

1. Existing diagnostic frameworks relying on standalone characterization techniques such as standalone NMR, UV–Vis, or ESI–MS are highly effective for tracking isolated single component chemical decomposition pathways. Nevertheless, they fundamentally fail to decouple the convoluted multicomponent cross interference occurring in real-world operational devices, such as free ligand penetration crossing the ion exchange membrane in alkaline iron organic chemistries, or the concurrent tracking of dead zinc detachment alongside competitive interfacial proton consumption (Fig. 8a). To transcend these boundaries, the field urgently requires the integration of high-resolution multi-modal operando characterization platforms capable of capturing coupled transient multi-physics fluctuations, bubble evolution, and active-site deactivation within electrode dead zones in three-dimensional space. As a promising forward-looking paradigm, implementing a coupled multi-modal approach such as integrated operando NMR and EPR can seamlessly track real-time valence shifts and radical intermediate formation to quantify the exact rates of chemical decomposition versus physical crossover. Crucially coupling these molecular level probes with synchrotron X-ray tomography enables the multi-modal visualization of macroscale transient fluidic fluctuations and structural electrode evolution across three-dimensional space with exceptional temporal resolution (Fig. 8b). Advanced AI algorithms, particularly physics-informed neural networks, can effectively bridge this diagnostic gap by seamlessly fusing these sparse experimental measurements such as online EIS or operando spectroscopy with core electrochemical transport governing equations. This physical mathematical synergy enables the high-fidelity tracking of elusive degradation roots including active species crossover, localized HER or OER trajectories, and electrolyte phase precipitation. Furthermore, real-time current density mapping facilitates the precise visualization of local reaction rates allowing researchers to decouple the discrete faradaic contributions of diffusion driven by concentration gradients and convection driven by pressure gradients during the transmembrane migration process. This unified multi-modal framework can simultaneously visualize the dynamic microscale bubble evolution from parasitic gas side reactions and map the spatial propagation of active-site deactivation directly within the hidden fluidic dead zones of highly porous electrodes. Correlating these real-time operando structural alterations with localized electrochemical signatures provides a definitive pathway to isolate transient mass transport bottlenecks and establish highly accurate predictive degradation models for long-duration stack operations.
2. Most art capacity recovery strategies remain predominantly passive and reactive, relying heavily on post degradation interventions such as manual hydraulic reflux, external chemical reduction via agents like oxalic acid, or manual cell swapping. However, these traditional valence regulation strategies suffer from insufficient reaction kinetics and prolonged system downtime when operating under aggressive grid conditions exceeding 200 mA cm^−2^, which ultimately culminates in incomplete capacity restoration (Fig. 8c). To break through these current kinetic bottlenecks and eliminate operational delays, the next paradigm shift must focus on autonomous in situ self-healing designs driven by advanced computational frameworks. Leveraging AI and machine learning for the high-throughput screening of responsive molecular reducing agents or smart membrane candidates offers a definitive pathway to accelerate recovery kinetics even under extreme current densities (Fig. 8d). This next-generation framework entails constructing smart responsive membranes capable of dynamically regulating ion transport to adaptively counter crossover-driven volume imbalances, while simultaneously introducing sacrificial chemical or catalytic rebalancing agents directly into the electrolyte matrix to eliminate state-of-charge deviations in real time without releasing external impurities or interrupting system operations.
3. Idealized mathematical models utilized for estimating SoH or monitoring autonomous operating schedules often diverge significantly from actual performance under complex grid-scale conditions due to the highly stochastic nature of nonlinear electrolyte degradation and multi-field coupled side reactions. While model-driven optimization has recently emerged, existing algorithms remain predominantly descriptive or rely heavily on black-box machine learning methodologies that lack fundamental physical constraints (Fig. 8e). To achieve multi-decade operational lifetimes, the ultimate frontier lies in embedding physics-informed neural networks into cloud-based digital-twin frameworks that seamlessly fuse real-time stack diagnostics with core multi-physics transport equations (Fig. 8f). At the molecular design, frontier-structured graph neural networks enable the high-throughput screening of chemically resilient RFBs materials by representing molecules as graphs and utilizing multi-layer message passing to predict thermodynamic properties like diffusion coefficients, oxidation potentials, and cleavage energies without costly density functional theory calculations. At the system scale, the microscale predictions directly feed into physics-informed neural networks that embed governing equations into their loss functions to achieve microsecond-level multi-physics state estimation. Furthermore, constructing this real-time digital-twin framework which mirrors the physical RFB stack via cloud computing platforms enables predictive diagnostics of the SoH and remaining useful life across multi-scale operating conditions. This digital physical synergy transitions the field from delayed maintenance to proactive predictive scheduling by leveraging deep reinforcement learning algorithms that operate on the digital-twin data stream to foresee imminent capacity imbalances before catastrophic failure occurs. Driven by these intelligent decisions-making algorithms, the system can autonomously execute closed-loop mitigation control and dynamically adjust operating boundary conditions.
4. Besides, current investigations largely rely on static standard potentials to evaluate parasitic side reactions within RFBs. However, the exact thermodynamic boundaries shift dynamically under localized concentration polarizations and asymmetric flow rates, leaving a critical research gap in mapping the transient thermodynamic excursions that drive local HER or OER competitions, especially under specific ligand coordination or localized pH drifts in Fe- and Zn-based RFB systems (Fig. 8g). This diagnostic challenge is further exacerbated under real-world grid scales where systems must endure harsh seasonal and diurnal temperature swings, yet current recovery protocols remain tailored exclusively for isothermal ambient conditions. Extreme temperatures trigger distinct, competing degradation pathways, where high temperatures accelerate active species deprotonation and catastrophic thermal precipitation such as V_2_O_5_ or MnO_2_, while low temperatures induce severe mass transport polarization due to exponential viscosity spikes. To transcend these fundamental boundaries, future work must establish quantitative phase diagrams that link flow field hydrodynamics with localized electrochemical stability windows, defining the precise operational envelopes that prevent irreversible active material precipitation from the root. Unlocking these thermal fluidic chemical coupling mechanisms represents a critical future frontier for establishing adaptive cross temperature capacity recovery protocols. Achieving this climate resilient capacity restoration across wide operational temperature windows requires the development of temperature responsive electrolyte additives that dynamically suppress hydroxyl condensation at elevated temperatures, alongside the deployment of localized hydrodynamic control algorithms to regulate compression ratios and flow rates in real time (Fig. 8h).

Looking ahead, with continuous breakthroughs in advanced in situ characterization technologies, and deep integration with front-end algorithms such as AI and big data prediction, it is expected to further deepen the understanding of RFBs capacity degradation mechanisms and develop more efficient capacity recovery strategies. These advancements will significantly prolong the cycle life of RFB systems and reduce full life-cycle operational maintenance costs as well as capacity degradation rates. Consequently, these improvements will facilitate the large-scale application of industrial-grade energy storage technologies characterized by long life and high stability for long-duration storage. Such progress provides essential support for the establishment of a green, low-carbon, flexible, and reliable new power system.
